# Single-Atom Catalysts Dispersed on Graphitic Carbon Nitride (g-CN): Eley–Rideal-Driven CO-to-Ethanol Conversion

**DOI:** 10.3390/nano15141111

**Published:** 2025-07-17

**Authors:** Jing Wang, Qiuli Song, Yongchen Shang, Yuejie Liu, Jingxiang Zhao

**Affiliations:** 1 College of Chemistry and Chemical Engineering, Harbin Normal University, Harbin 150025, China; wjhebsfdx@stu.hrbnu.edu.cn (J.W.); hsdsql@stu.hrbnu.edu.cn (Q.S.); 2 Modern Experiment Center, Harbin Normal University, Harbin 150025, China; liuyuejie@hrbnu.edu.cn

**Keywords:** CO electroreduction, single-atom catalysts, graphitic carbon nitride, multi-carbon productions, density functional theory computations

## Abstract

The electrochemical reduction of carbon monoxide (COER) offers a promising route for generating value-added multi-carbon (C_2+_) products, such as ethanol, but achieving high catalytic performance remains a significant challenge. Herein, we performed comprehensive density functional theory (DFT) computations to evaluate CO-to-ethanol conversion on single metal atoms anchored on graphitic carbon nitride (TM/g–CN). We showed that these metal atoms stably coordinate with edge N sites of g–CN to form active catalytic centers. Screening 20 TM/g–CN candidates, we identified V/g–CN and Zn/g–CN as optimal catalysts: both exhibit low free-energy barriers (<0.50 eV) for the key ^*^CO hydrogenation steps and facilitate C–C coupling via an Eley–Rideal mechanism with a negligible kinetic barrier (~0.10 eV) to yield ethanol at low limiting potentials, which explains their superior COER performance. An analysis of d-band centers, charge transfer, and bonding–antibonding orbital distributions revealed the origin of their activity. This work provides theoretical insights and useful guidelines for designing high-performance single-atom COER catalysts.

## 1. Introduction

The electrochemical reduction of carbon dioxide (CO_2_ER) provides an attractive route for addressing global energy and environmental challenges by producing value-added multi-carbon (C_2+_) hydrocarbons and oxygenates using renewable electricity [1,2,3]. However, the direct conversion of CO_2_ to C_2+_ products via CO_2_ER is significantly hindered by poor CO_2_ solubility in alkaline electrolytes, where carbonate formation occurs, leading to low overall efficiency [4]. To overcome these limitations, a cascade reduction strategy has been proposed, involving a two-step pathway: first, converting CO_2_ to CO with high selectivity (>90%); then, reducing CO to C_2+_ products via CO electroreduction (COER) [5,6,7]. Compared to CO_2_ER, COER holds great promise for achieving high conversion efficiency to C_2+_ products at low cell voltages due to the following advantages [8,9,10,11]: (1) CO is inert toward alkaline electrolytes, preventing carbonate formation. (2) The alkaline environment suppresses the competing hydrogen evolution reaction (HER), enhancing selectivity. (3) CO possesses higher chemical reactivity than CO_2_, improving the overall conversion efficiency. (4) As a key intermediate in the CO_2_ER network, studying the COER mechanism provides deeper insights into CO_2_ER processes.

Catalysts are essential for improving both activity and selectivity in catalytic reactions. For COER, copper (Cu) catalysts have been extensively utilized to generate C_2+_ products [12,13,14,15,16]. However, the critical C–C coupling step in COER-derived C_2+_ formation is strongly influenced by the surface structures of Cu catalysts. In this regard, C–C coupling between two adsorbed ^*^CO molecules occurs readily on Cu(100) and Cu(110) surfaces, whereas the Cu(111) facet shows poor performance for this step [17,18,19,20]. Moreover, the C–C coupling on these facets typically follows a Langmuir–Hinshelwood (L-H) mechanism, necessitating high CO coverage. Unfortunately, Cu binds ^*^CO relatively weakly, whereas metals like Pt bind CO too strongly, resulting in CO poisoning [21]. Consequently, designing catalysts that enable efficient C–C coupling via alternative pathways is critical for enabling the selective COER to C_2+_ products.

In this context, single-atom catalysts (SACs) have garnered significant attention due to their high atomic utilization efficiency and exceptional catalytic performance in various electrochemical reactions [22,23,24,25,26], including COER for producing C_2+_ products [27,28,29]. For instance, Wang et al. demonstrated that a single Cu atom exhibited remarkable activity for COER, yielding C_2+_ products, with acetate being the predominant product, achieving a Faradaic efficiency (FE) of approximately 50% [27]. Additionally, Bao et al. reported that single Cu atoms anchored on Ti_3_C_2_T_x_ MXene nanosheets served as efficient catalysts for CO electroreduction, achieving an ethylene formation selectivity of 71% [28]. Moreover, Miao et al. proposed that isolated Cu atoms coordinated with N and P atoms could serve as promising COER catalysts for acetate production in alkaline media, demonstrating a selectivity of 63.9% [29]. Theoretically, Keitaro et al. revealed that single Cu atoms supported on covalent triazine frameworks could significantly enhance the electrochemical conversion of CO to ethanol, overcoming sluggish C–C coupling with a kinetic barrier of less than 0.1 eV [30].

Building on the pioneering studies of COER catalyzed by single Cu catalysts, an important question emerges: Can other SACs also be employed for CO electroreduction to C_2+_ products? If so, which single metal demonstrates superior catalytic performance, and what underlies its catalytic activity? To address these questions, we conducted a systematic investigation of SACs anchored on graphitic carbon nitrides (g–CN) as representative catalysts for CO electroreduction to C_2+_ products, employing comprehensive density functional theory (DFT) calculations. Notably, the g–CN layer has emerged as an ideal support for anchoring SACs due to its uniform hole vacancies, exceptional thermal and chemical stability, abundant edge sp^2^-bonded nitrogen sites with lone-pair electrons, and widespread validation in both experimental [31] and DFT investigations of SACs [32,33,34].

The results revealed that the electronic charges of these SACs play a decisive role in CO activation, with Sc, Ti, V, Zn, and Zr catalysts requiring lower energy inputs (<0.50 eV) to drive the hydrogenation of activated ^*^CO to the ^*^CHO intermediate. Moreover, anchored V and Zn atoms were identified as promising COER catalysts owing to their low kinetic barrier (approximately 0.10 eV) for C–C coupling between ^*^CHO and CO molecules via the Eley–Rideal mechanism, resulting in the efficient generation of ethanol at low limiting potentials.

## 2. Computational Methods and Models

All spin-polarized density functional theory (DFT) calculations were performed using the Perdew–Burke–Ernzerhof (PBE) [35] functional within the generalized gradient approximation (GGA) according to our test, as displayed in Table S1, implemented in the Vienna ab initio Simulation Package (VASP) [36,37], which is regarded as one of the most commonly used functionals in surface science and catalysis. Due to the considerable computational cost of hybrid functionals such as HSE06, particularly for the 20 large-scale TM/g–CN systems consisting of 49 atoms, all electronic structure calculations were performed using the PBE functional based on our tests (Figure S1), which has been widely validated for similar SAC systems in the literature [32,33,34]. The projector-augmented wave (PAW) [38,39] method was utilized to describe the interactions between electrons and ions, employing a plane-wave cutoff of 550 eV. Van der Waals interactions between adsorbates and catalysts were accounted for using the DFT-D3 method within Grimme’s scheme [40] according to our test (Table S1). Convergence criteria of 1 × 10^−5^ eV for total energy and 0.01 eV Å^−1^ for force were applied. Furthermore, a 2 × 2 × 1 supercell of g–CN was constructed with a 20 Å vacuum layer to minimize interlayer interactions. During structural optimization, a 3 × 3 × 1 Monkhorst–Pack k-point grid was employed, while a finer grid of 7 × 7 × 1 was used for electronic property calculations. Bader charge analysis was conducted to determine the involved charges, and ab initio molecular dynamics (AIMD) simulations [41] were performed at 300 K for 10 and 20 ps to assess the kinetic stability of the SACs, which is consistent with standard practice for systems containing light atoms such as H, C, N, and transition metals. This timestep allows for the accurate integration of atomic trajectories and reliable description of vibrational motions, particularly for high-frequency lattice modes. The CI-NEB method was employed to calculate the kinetic barrier for C–C coupling [42].

To evaluate the catalytic performances of these SACs for COER from a thermodynamic perspective, the Gibbs free energy changes (ΔG) for all possible elementary steps were computed using the following computational hydrogen electrode (CHE) model [43,44]: ∆G=∆E+∆ZPE−T∆S. Here, ∆E is the reaction energy derived from DFT computations. For example, the reaction energy of CO adsorbed on the TM/g–CN was defined as ∆E = *E*_CO–TM/g–CN_–*E*_TM/g–CN_–*E*_CO_, where *E*_CO–TM/g–CN_, *E*_TM/g–CN_, and *E*_CO_ represent the total electronic energies for the CO adsorbed on the TM/g–CN, free TM/g–CN, and CO molecule, respectively.∆ZPE and ∆S represent the correction of zero-point energy and entropy, respectively. It should be noted that the ZPE and S of isolated molecules (such as CO, H_2_, and H_2_O) were sourced from the NIST database, while those of the adsorbed intermediates were computed based on their vibrational frequencies. Then, the limiting potential (U_L_), a crucial indicator for evaluating catalytic activity, was computed from the obtained ∆*G* values as U_L_ = −∆*G_max_*/e, where ∆*G_max_* is the maximum free energy change for all elementary steps. A less negative U_L_ indicates a lower applied potential required for the potential-determining step (PDS), thus reflecting higher catalytic activity toward COER.

## 3. Results and Discussion

### 3.1. Screening of SACs Candidates

**Figure 1:**
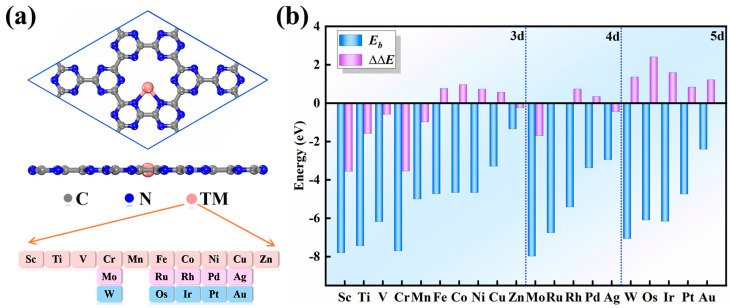
(**a**) The considered SACs anchored on g–CN, where the gray, blue, and pink balls represent the C, N, and TM atoms, respectively, and (**b**) their computed *E*_bind_ and ∆∆*E* values.

Before screening SAC candidates, we first examined their configuration and stability on the g–CN support, including 3d (Sc ~ Zn), 4d (Mo, Ru, Rh, Pd, and Ag), and 5d (W, Os, Ir, Pt, and Au), as shown in Figure 1a. After geometric optimization, the structure of the g–CN substrate underwent minimal structural changes upon metal anchoring, as its large cavity was sufficient to accommodate a single metal atom. For the pristine g-CN monolayer, we performed phonon dispersion calculations using density functional perturbation theory (DFPT) to assess its stabilization. As shown in Figure S2, the phonon spectrum exhibited no imaginary frequencies, confirming the dynamical stability of the g-CN support. In contrast, we note that some imaginary frequencies were observed for a specific allotrope of the c-C_3_N_2_ system, indicating that its particular structure may have dynamical instability [45]. After metal anchoring on g-CN substrate, these metals formed coordinated bonds with two or three N atoms around the hole of the g–CN support, with bond lengths ranging from 1.86 to 2.54 Å. Although the N atoms coordinating the metal exhibit slightly elongated N–C bond lengths with their neighboring C atoms (Figure S3), the overall g–CN monolayer showed no significant distortion. To evaluate the stability of these anchored single metal atoms, we computed their binding energies (*E*_bind_) according to the following equation: $E_{bind}=E_{TM/g-CN}-E_{g-CN}-E_{TM}\;$. Here, $E_{TM/g-CN}$, $E_{g-CN}$, and $E_{TM}$ are the electronic energies of the adsorbed TM atoms, pristine g–CN, and isolated TM atoms, respectively. As shown in Figure 1b, all studied TM atoms exhibited rather negative *E*_b_ values (−1.35~−7.97 eV), suggesting their good thermodynamic stability. Notably, the above results regarding the configurations and stabilities of the anchored metals are well consistent with those of previous theoretical studies [46,47,48,49]. In addition, the feasibility of metal atom aggregation was assessed by computing the difference (∆∆*E*) between *E*_bind_ and cohesive energies (*E*_coh_) as ∆∆*E* = *E*_bind_ − *E*_coh_, where a negative ∆∆*E* value indicates resistance to aggregation. Notably, *E*_coh_ was determined by *E*_coh_ = *E*_bulk_/n, where *E*_bulk_ is the computed total energies of the bulk metal and n is the number of atoms in the metallic bulk materials. According to this criterion, Fe, Co, Ni, Cu, Rh, Pd, W, Os, Ir, Pt, and Au are prone to aggregation into larger clusters due to their positive ∆*E* values (Figure 1b). To comprehensively understand the trends in CO adsorption and activation, however, these candidates were retained for further comparison. The strong anchoring behavior of metal atoms on g–CN substrate induced substantial charge transfer from the metal to the substrate (0.53~1.68, Table S2), endowing the anchored single metal atom with variable positive charge. In addition, due to the unpaired d electrons within metal atoms, most of the TM/g–CN materials had significant magnetic moments, which were mainly localized on the metal atoms (Table S2). Consequently, the considerable positive charge and magnetic moment made the anchored single metal atoms the catalytically active sites in COER.

Regardless of the reaction mechanism and products, CO adsorption and activation are the initial and essential steps in CO electroreduction [50]. Therefore, the CO adsorption strength, quantified by its free adsorption energy (∆G_*CO_), on these SAC candidates was employed as the primary criterion for screening efficient COER catalysts. Furthermore, as ^*^CO hydrogenation is widely recognized as another vital step (the most likely PDS) in COER, it was chosen as the second screening criterion, requiring a minimal free energy change (<0.50 eV).

**Figure 2:**
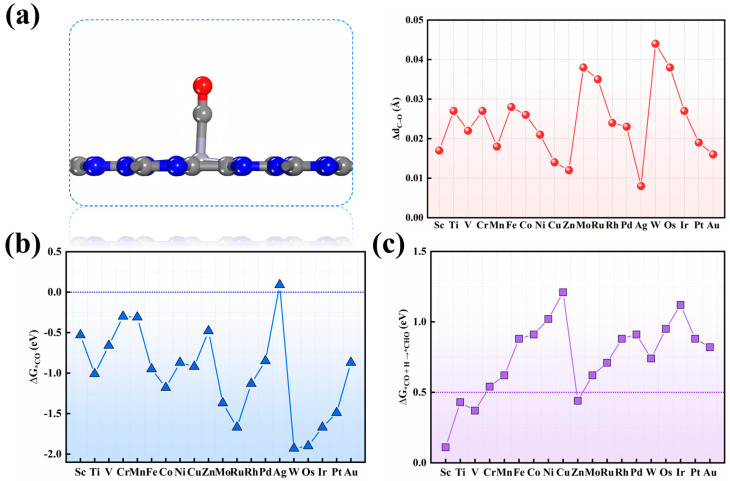
(**a**) The optimized adsorption configuration of the CO molecule on TM/g–CN candidates and the corresponding changes in the C–O bond length before and after CO adsorption. Gray, blue, and red balls represent C, N, and O atoms, respectively. (**b**) The computed free adsorption energies of CO (∆G_*CO_) and (**c**) ^*^CO hydrogenation to ^*^CHO species on these TM/g–CN candidates.

Anchored metal sites, characterized by positive charges and partially filled d-orbitals, bind CO molecules through electron donation–acceptance interactions, thus serving as active sites for CO adsorption. It should be noted that CO adsorption was only considered via the carbon atom (Figure 2a) due to the well-established donation/back-donation mechanism. To validate this preference, we additionally computed the adsorption energy of CO via the O atom on selected TM/g-CN systems. For example, on V/g-CN, the adsorption energy of C-end CO is −0.66 eV, while that of O-end CO is −0.31 eV, making the former significantly more favorable. Similar trends were found on Zn/g-CN (−0.48 eV vs. −0.15 eV). These results indicated that C-end binding is energetically more stable by 0.30–0.50 eV across representative systems, confirming that the CO–M interaction via the C atom is preferred due to stronger orbital hybridization (σ donation from C and π back-donation from metal d orbitals). Furthermore, except for Ag, all SAC candidates exhibit strong CO adsorption (∆G_*CO_ = −0.30 to −1.93 eV; Figure 2b), resulting in C–O bond elongations that vary by about 0.70%~3.86% (Table S3) that reflect different activation extents. Thus, under the first screening criterion, only Ag is excluded due to its weak CO adsorption. The remarkable differences in CO adsorption among the various candidates potentially arise from the different d-electrons, charges, d-band centers, and magnetic moments of these anchored metal sites. Which of these factors predominates will be elucidated in future work using machine learning techniques. Applying the second criterion, we computed the free energy change (∆*G*) for hydrogenating activated ^*^CO to ^*^CHO (or ^*^COH). The computations indicated that activated ^*^CO preferentially hydrogenates to ^*^CHO on all SACs. The free energy changes for the ^*^CO → ^*^CHO step ranged from 0.11 to 1.21 eV (Figure 2c), among which Sc, Ti, V, and Zn exhibit ∆*G* < 0.50 eV, identifying them as highly promising COER catalysts, while other SACs are ruled out. Furthermore, according to our DFT computations on the free energy diagrams, Sc and Ti candidates were also excluded as promising COER catalysts due to their excessively strong adsorption of the reaction products (Figure S4). Consequently, only V and Zn catalysts were selected as promising COER candidates.

### 3.2. Product Distribution and Reaction Mechanism

After identifying V and Zn as promising COER catalysts, we investigated the complete COER pathway to determine the lowest energy pathway on the two catalysts, which was defined as the pathway with the lowest positive elementary free energy change between any two steps. Along the lowest energy pathway, the needed applied voltage was least negative when the whole reaction became exergonic.

Following ^*^CHO formation on both catalysts, we first investigated the pathway for C_1_ product formation. Specifically, hydrogenation of ^*^CHO at the carbon or oxygen site yielded ^*^CH_2_O or ^*^CHOH species, respectively. Computing Δ*G* for both hydrogenation pathways revealed that conducting carbon-site hydrogenation to form ^*^CH_2_O was more energetically favorable than conducting oxygen-site hydrogenation to form ^*^CHOH. The computed Δ*G* values for ^*^CH_2_O formation were −0.57 eV on V and 0.48 eV on Zn catalysts, respectively. Next, adding the third (H^+^ + e^−^) pair yielded either ^*^CH_3_O or ^*^CH_2_OH intermediates. DFT results show that ^*^CH_3_O formation was preferred, with Δ*G* values of −0.61 eV (V) and −1.27 eV (Zn), compared to −0.03 eV and −0.83 eV for ^*^CH_2_OH formation (Table S4). In the subsequent hydrogenation step on V, strong interactions with the ^*^O group (*E*_ads_ = −6.67 eV) favored CH_4_ formation over CH_3_OH generation by 1.39 eV. In contrast, weaker interaction between the Zn active site and ^*^O species (*E*_ads_ = −3.62 eV) made CH_3_OH formation more favorable than CH_4_ by 1.92 eV.

**Figure 3:**
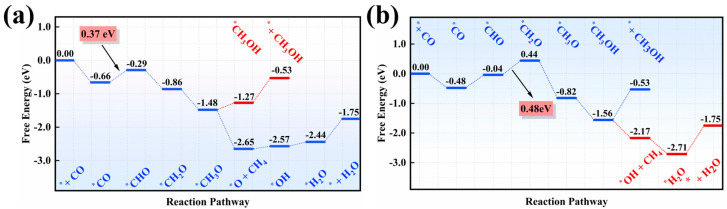
The most favorable free energy profiles of COER for C_1_ products on the (**a**) V/g–CN and (**b**) Zn/g–CN catalysts.

Overall, from the computed ∆G values for each elementary reaction in the C_1_ generation pathway, we predicted that the domain C_1_ products in COER on single V and Zn catalysts were CH_4_ and CH_3_OH, respectively, with ∆G_max_ values of 0.37 eV (^*^CHO formation) and 0.48 eV (^*^CH_2_O formation), as shown in Figure 3, while the corresponding intermediates are presented in Figure S5. Accordingly, U_L_ was computed as −0.37 V on V/g–CN and −0.48 V on Zn/g–CN, respectively.

**Figure 4:**
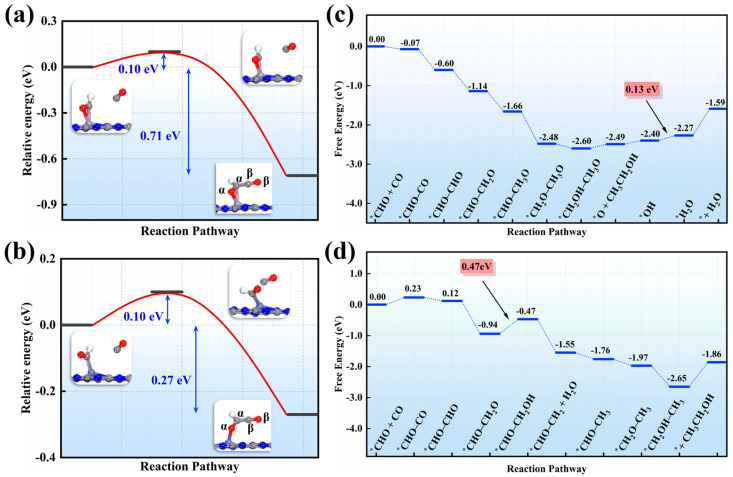
The computed kinetic processes for the C–C coupling between CO molecules and ^*^CHO intermediates on (**a**) V/g–CN and (**b**) Zn/g–CN catalysts and the most favorable free energy profiles for C_2_H_5_OH production on (**c**) V/g–CN and (**d**) Zn/g–CN catalysts.

Compared to C_1_ products, we are more interested in C_2_ products on these two SAC candidates. Previous studies identify two C–C coupling pathways: (1) CO dimerization [51]; (2) coupling of unsaturated C-based intermediates (e.g., ^*^CHO, ^*^CH_2_) formed in the C_1_ pathway with CO [52,53]. For pathway 1, our DFT results show that conducting CO dimerizetion to form the ^*^C_2_O_2_ precursor is highly unfavorable (Δ*G* = 0.84 eV on V, 0.65 eV on Zn, Figure S6), so it is, therefore, excluded for forming C_2_ products. In pathway 2, C-containing intermediates are crucial for C–C coupling. Among the C-based species in the C_1_ pathway, ^*^CHO (with an unsaturated C site) was chosen for coupling with CO. Since V and Zn SACs each provide a single active site, ^*^CHO couples with CO via an Eley–Rideal (E–R) mechanism. Remarkably, the kinetic barrier for ^*^CHO–CO coupling is as low as ~0.10 eV (Figure 4a,b), accompanied by exothermic releases of 0.71 eV (V) and 0.27 eV (Zn), indicating that this E–R–mediated C–C coupling is both thermodynamically and kinetically favorable.

After establishing ^*^CHO–CO coupling, we examined the subsequent hydrogenation pathways leading to various C_2_ products. To distinguish the two different C and O atoms, the key ^*^CHO–CO intermediate was relabeled as C_α_HO_α_–C_β_O_β_. For clarity, the single-atom V catalyst was chosen as a representative to elucidate the complete hydrogenation pathway of ^*^C_α_HO_α_–C_β_O_β_ hydrogenation to C_2_ products. Based on computing ∆G values for all possible elementary steps, the most energetically favorable pathway of ^*^C_α_HO_α_–C_β_O_β_ hydrogenation is summarized in Figure 4c,d, with the corresponding intermediates shown in Figure S5.

Specifically, in this optimal pathway, the C_β_ atom of ^*^C_α_HO_α_–C_β_O_β_ is first hydrogenated to yield the ^*^C_α_HO_α_–C_β_HO_β_ intermediate (∆*G* = −0.53 eV), which is more favorable than alternative hydrogenation products (Table S5), including ^*^C_α_H_2_O_α_–C_β_O_β_ (−0.34 eV). ^*^C_α_HO_α_–C_β_O_β_H (0.03 eV), and ^*^C_α_HO_α_H–C_β_O_β_ (0.43 eV). Subsequently, further hydrogenation at the C_β_ atom produces ^*^C_α_HO_α_–C_β_H_2_O_β_ and ^*^C_α_HO_α_–C_β_H_3_O_β_ species, with ∆*G* values of −0.54 eV and −0.52 eV, respectively. Notably, both intermediates feature the simultaneous adsorption of both O atoms onto the single V active site. Additionally, cleavage of the C_β_–O_β_ bond in ^*^C_α_HO_α_–C_β_H_3_O_β_ form ^*^C_α_HO_α_C_β_H_3_…O_β_ denotes the broken bond. In the next step, hydrogenation at C_α_ produces the ^*^C_α_H_2_O_α_C_β_H_3_…O_β_ intermediate, which is exergonic by 0.82 eV. The hydrogenation of O_α_ leads to the formation of the adsorbed C_2_H_5_OH product (Δ*G* = −0.12 eV), which desorbs from the V site after surmounting a small free energy barrier of 0.12 eV. Finally, the remaining ^*^O species is reduced to H_2_O via two successive hydrogenation steps.

Overall, throughout the complete C_2_ product formation pathway, every hydrogenation step of the carbon-containing intermediates is exergonic. In other words, ^*^CHO formation in the C_1_ pathway remains the potential-determining step for C_2_H_5_OH production. Similarly, ethanol can be yielded on the single-atom Zn catalyst with a U_L_ of −0.47 V (Figure 4d).

Another critical consideration is catalyst selectivity. Since the hydrogen evolution reaction (HER) competes with CO electroreduction [54,55], we assessed the HER performances of both catalysts by calculating the adsorption free energy of the ^*^H intermediate. The results indicate that, owing to electrostatic repulsion between H^+^ and the positively charged active sites, ^*^H adsorption is 0.24 eV on V and −0.35 eV on Zn (Figure S7), weaker than CO adsorption (−0.66 and −0.48 eV), demonstrating that both catalysts preferentially bind CO over ^*^H and, thus, exhibit high selectivity for COER.

### 3.3. V/g–CN and Zn/g–CN of COER Activity Origin

**Figure 5:**
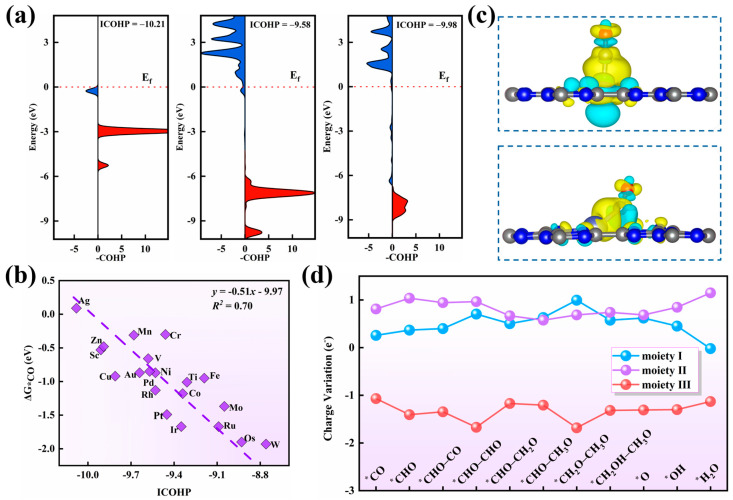
(**a**) The computed crystal orbital Hamiltonian population (COHP) of CO before and after being adsorbed on V/g–CN and Zn/g–CN surfaces. (**b**) The variation in CO free adsorption energy and the integral-crystal orbital Hamiltonian population (ICOHP) of the formed C–O bond. (**c**) The charge density difference of the CO molecule on the V/g–CN catalyst, where the isosurface value is set to be 0.001 e Ǻ^−3^, and cyan and yellow bubbles represent positive and negative charges, respectively. (**d**) The charge variation during the COER reaction on the V/g–CN surface. Moiety Ⅰ, Ⅱ, and Ⅲ represent the adsorbed intermediates, TM–N_2_, and g–CN substrate, respectively.

To gain deeper insight into the superior COER performances of these catalysts, we performed the integrated crystal orbital Hamiltonian population (ICOHP) analyses on adsorbed CO, where more negative ICOHP values indicate stronger C–O bonds and, thus, reduced CO activation. Upon adsorption on V and Zn catalysts, CO exhibits several antibonding states near the Fermi level (Figure 5a), indicating strong catalyst–CO interactions and effective activation, as confirmed by less negative ICOHP values (−9.58 on V and −9.98 on Zn) than that of the free CO molecule (−10.21). Notably, a linear correlation between ICOHP and CO adsorption free energy (Figure 5b) demonstrates that variations in CO adsorption strength across metals stem from differences in bonding and antibonding orbital populations. Strong CO adsorption is further supported by significant charge transfer from the V and Zn catalysts to CO (0.26 and 0.10 e, respectively), consistent with the computed charge density difference maps (Figure 5c). In addition, we analyzed charge evolution along the CO–to–C_2_H_5_OH reaction pathway by partitioning each adsorbed intermediate into three components: the adsorbed C_x_H_y_O_z_ intermediates (moiety I), metal and the two coordinated N atoms (moiety II), and the g–CN support (moiety III, Figure S8). Taking V/g–CN as an example, we found that the V–N_2_ moiety always gains charge and nearly remains unchanged during the whole COER process (Figure 5d). Furthermore, the g–CN substrate can be regarded as an electron reservoir, which may provide electrons to the V–N_2_ transmitter, and it then transfers them to the adsorbed C_x_H_y_O_z_ intermediates on the surface.

Although phonon spectrum calculations can indeed provide valuable insights into the dynamical stability of catalytic systems, the TM/g–CN catalysts and their representative reaction intermediates investigated in this study typically contain more than 50 atoms, including heavy transition metal centers and multiple adsorbed species. Performing full phonon calculations for each of these large systems—particularly using finite-difference methods—would require prohibitively high computational resources. Therefore, considering the high-throughput nature and scope of this DFT screening study, such calculations were not carried out. Instead, AIMD simulations for 10 and 20 ps were employed to confirm the stabilities of the two catalysts. The results demonstrated that the total energies of these two systems fluctuate minimally throughout the AIMD process, and there is no significant distortion or deformation in their geometric configurations (Figure S9). Remarkably, throughout the AIMD process, the TM–N chemical bonds remain intact, with bond lengths varying between approximately 1.80 and 2.40 Å (Figure S10), again indicating that these two materials have good stability and can be used as COER catalysts. In addition, we examined the mechanical stability of V/g–CN and Zn/g–CN. As shown in Table S6, we found that the elastic constants of both materials satisfy the Born criteria (C_11_C_22_ − C_12_^2^ > 0 and C_66_ > 0), indicating their excellent mechanical stability.

## 4. Conclusions

In summary, through comprehensive DFT calculations, we evaluated the potential of various SACs on g–CN nanosheets as COER catalysts for C_2_ product formation. Based on CO activation and hydrogenation metrics, single-atom V and Zn catalysts were identified as highly effective COER catalysts, featuring small limiting potentials (<−0.50 V), low C–C coupling barriers (~0.10 eV), and strong suppression of HER. Their exceptional activity stems from balanced bonding and antibonding orbital distributions at the active sites, enabling optimal CO activation. We hope that this work inspires further experimental and theoretical efforts to deploy SACs for CO electroreduction toward diverse high-value products.

## Data Availability

Data are contained within the article and Supplementary Materials.

## Supplementary Materials

The following supporting information can be downloaded at: https://www.mdpi.com/article/10.3390/nano15141111/s1, Table S1: (a) The computed adsorption energy (*E_ads_*, eV) of CO on V/g–CN and Zn/g–CN catalysts, limiting potential (*U_L_*, V) for COER, and the energy barrier (*E_barr_*, eV) for C–C coupling; (b) The computed adsorption energy (*E_ads_*, eV) of CO on V/g–CN and Zn/g–CN catalysts; Table S2: The formed metal–N bond lengths (*d*_M–N_, Å), charge transfer from the anchored single metal atoms to the g–CN substrates (*Q*, |e^−^|), the computed binding energies (*E*_bind_, eV), the magnetic moments (*μ*, μ_B_) of TM/g–CN; Table S3: The computed free energy of CO adsorption (∆*G_*CO_*, eV), formed C–O bond lengths (*d*_C_–_O_, Å), percentage elongation of the C–O bond length (*Bond elongation*, %); Table S4: The computed free energy changes (Δ*G*, eV) of all potential elementary steps from COER to C_1_ products on V/g–CN and Zn/g–CN catalysts. The most favorable steps were marked in red; Table S5: The computed free energy changes (Δ*G*, eV) of all potential elementary steps from COER to C_2_ products on V/g–CN and Zn/g–CN catalysts. The most favorable steps were marked in red; Table S6: Elastic constants (*C_11_*, *C_22_*, *C_12_*, and *C_66_*, in N m^−1^) and Young’s modulus (*Y_x_*, *Y_y_*: the Y value along the x, y direction, in N m^−1^); Figure S1; The projected density of states (PDOS) for CO adsorption is presented for (a) V/g–CN and (b) Zn/g–CN catalysts using the PBE functional, as well as for (c) V/g–CN and (d) Zn/g–CN catalysts using the HSE06 functional; Figure S2: The computed phonon spectrum of pristine g-CN monolayer; Figure S3: The variation of N–C bond lengths between metal-coordinated nitrogen atoms and their adjacent carbon atoms; Figure S4: The most favorable free energy profiles for COER to C_1_ products on the (a) Sc/g–CN and (b) Ti/g–CN catalysts, and to C_2_H_5_OH production on (c) Sc/g–CN and (d) Ti/g–CN catalysts; Figure S5: The corresponding intermediates involved in the lowest energy path of the COER to produce C_1_ products on (a) V/g–CN and (b) Zn/g–CN catalysts; Figure S6: The corresponding intermediates involved in the lowest energy path of the COER to produce C_2_ products on (a) V/g–CN and (b) Zn/g–CN catalysts; Figure S7: The hydrogen evolution reaction on V/g–CN and Zn/g–CN catalysts; Figure S8: Three adsorbed ^*^C_x_H_y_O_z_ intermediates on V/g–CN, which was divided into three moieties: moiety Ⅰ: absorbed molecules; moiety Ⅱ: TM–N_2_; and moiety Ⅲ: g–CN substrate); Figure S9: Temperature and energy changes over time in the AIMD simulation of (a) (c) V/g−CN and (b) (d) Zn/g−CN catalysts. The simulations were performed for 10 ps and 20 ps at 300 K, respectively; Figure S10: TM–N bond length over time in the AIMD simulation of (a) V/g–CN and (b) Zn/g–CN catalysts. The simulations were performed for 10 ps at 300 K.
