# Supplementary material for: Single-Atom Catalysts Dispersed on Graphitic Carbon Nitride (g-CN): Eley–Rideal-Driven CO-to-Ethanol Conversion
Source: Nanomaterials (Basel). 2025 Jul 17;15(14):1111. doi: 10.3390/nano15141111 (PMC12299792; doi:10.3390/nano15141111)
Supplement: Supplementary file 1 [file nanomaterials-15-01111-s001.zip › nanomaterials-3723153-supplementary.docx]

**Supporting Information**

**Single-Atom Catalysts Dispersed on Graphitic Carbon Nitride (g−CN):**

**Eley–Rideal-Driven CO‑to‑Ethanol Conversion**

*Jing Wang,^a,#^ Qiuli Song,^a,#^ Yongchen Shang^a,*^ Yuejie Liu,^b^ Jingxiang Zhao^a,*^*

*^a^* *College of Chemistry and Chemical Engineering, Harbin Normal University, Harbin, 150025, China*

*^b^ Modern Experiment Center, Harbin Normal University, Harbin, 150025, China.*

*^*^ To whom correspondence should be addressed. Email: [yongchenshang@163.com](mailto:yongchenshang@163.com) (Y. S.); zhaojingxiang@hrbnu.edu.cn (J.Z.)*

*^#^ Jing Wang and Qiuli Song contributed equally to this work.*

**Table S1.** (a) The computed adsorption energy (*E_ads_*, eV) of CO on V/g−CN and Zn/g−CN catalysts, limiting potential (*U_L_*, V) for COER, and the energy barrier (*E_barr_*, eV) for C−C coupling.

|  | *E_ads_* | | *U_L_* | | *E_barr_* | |
| --- | --- | --- | --- | --- | --- | --- |
|  | V/g−CN | Zn/g−CN | V/g−CN | Zn/g−CN | V/g−CN | Zn/g−CN |
| PBE | –1.32 | –1.14 | 0.37 | 0.48 | 0.10 | 0.10 |
| RPBE | –1.22 | –0.94 | 0.43 | 0.46 | 0.01 | 0.12 |

(b) The computed adsorption energy (*E_ads_*, eV) of CO on V/g−CN and Zn/g−CN catalysts.

|  | *E_ads_* | |
| --- | --- | --- |
|  | V/g−CN | Zn/g−CN |
| DFT-D3 | –1.32 | –1.14 |
| optB88-vdw | –1.28 | –1.11 |

**Table S2.** The formed metal−N bond lengths (*d*_M−N_, Å), charge transfer from the anchored single metal atoms to the g−CN substrates (*Q*, |e^-^|), the computed binding energies (*E*_bind_, eV), the magnetic moments (*μ*, μ_B_) of TM/g−CN.

|  | *dTM_–_N* | *E*_bind_ | *Q* | *μTM/μTM/g*_–_*CN* |
| --- | --- | --- | --- | --- |
| Sc/g−CN | 2.13 | –7.80 | –1.68 | 0.32/0.66 |
| Ti/g−CN | 2.04 | –7.43 | –1.36 | 1.57/1.61 |
| V/g−CN | 1.96 | –6.17 | –1.18 | 2.81/2.65 |
| Cr/g−CN | 1.94 | –7.69 | –1.22 | 3.61/3.45 |
| Mn/g−CN | 2.08 | –5.00 | –1.24 | 4.32/4.36 |
| Fe/g−CN | 2.00 | –4.72 | –0.91 | 3.30/3.59 |
| Co/g−CN | 1.93 | –4.66 | –0.93 | 2.04/1.47 |
| Ni/g−CN | 1.92 | –4.66 | –0.76 | 1.02/1.57 |
| Cu/g−CN | 2.04 | –3.29 | –0.71 | 0.02/0.40 |
| Zn/g−CN | 1.97 | –1.35 | –1.12 | 0.00/0.00 |
| Mo/g−CN | 2.00 | –7.97 | –1.21 | 2.65/1.92 |
| Ru/g−CN | 1.86 | –6.75 | –0.85 | 0.09/0.07 |
| Rh/g−CN | 1.88 | –5.41 | –0.62 | 0.06/0.33 |
| Pd/g−CN | 2.08 | –3.37 | –0.60 | –0.76/1.38 |
| Ag/g−CN | 2.54 | –2.95 | –0.67 | 0.00/0.00 |
| W/g−CN | 1.97 | –7.06 | –1.28 | 2.45/1.88 |
| Os/g−CN | 1.86 | –6.09 | –0.98 | 0.00/0.00 |
| Ir/g−CN | 1.87 | –6.16 | –0.69 | 0.08/0.40 |
| Pt/g−CN | 1.91 | –4.74 | –0.71 | 0.00/0.00 |
| Au/g−CN | 2.44 | –2.40 | –0.53 | 0.00/0.00 |

**Table S3.** The computed free energy of CO adsorption (*∆G_*CO_*, eV), formed C−O bond lengths (*d*_C−O_, Å), percentage elongation of the C−O bond length (*Bond elongation*, %).

|  | *∆G_*CO_* | [*d_C_*_−_*_O_*](mailto:ETM@g-CN) | *Bond elongation* |
| --- | --- | --- | --- |
| Sc/g−CN | –0.53 | 1.16 | 1.49% |
| Ti/g−CN | –1.01 | 1.17 | 2.37% |
| V/g−CN | –0.66 | 1.16 | 1.93% |
| Cr/g−CN | –0.30 | 1.17 | 2.37% |
| Mn/g−CN | –0.31 | 1.16 | 1.58% |
| Fe/g−CN | –0.95 | 1.17 | 2.46% |
| Co/g−CN | –1.18 | 1.17 | 2.28% |
| Ni/g−CN | –0.87 | 1.16 | 1.84% |
| Cu/g−CN | –0.92 | 1.15 | 1.23% |
| Zn/g−CN | –0.48 | 1.15 | 1.05% |
| Mo/g−CN | –1.37 | 1.18 | 3.33% |
| Ru/g−CN | –1.67 | 1.17 | 3.07% |
| Rh/g−CN | –1.13 | 1.16 | 2.11% |
| Pd/g−CN | –0.85 | 1.16 | 2.02% |
| Ag/g−CN | 0.09 | 1.15 | 0.70% |
| W/g−CN | –1.93 | 1.18 | 3.86% |
| Os/g−CN | –1.90 | 1.18 | 3.33% |
| Ir/g−CN | –1.67 | 1.17 | 2.37% |
| Pt/g−CN | –1.49 | 1.16 | 1.67% |
| Au/g−CN | –0.87 | 1.16 | 1.40% |

**Table S4.** The computed free energy changes (*ΔG*, eV) of all potential elementary steps from COER to C_1_ products on V/g−CN and Zn/g−CN catalysts. The most favorable steps were marked in red.

| V/g−CN | *∆G* |
| --- | --- |
| ^*^ + CO → ^*^CO | –0.66 |
| ^*^CO + H^+^ + e^-^ →^*^CHO | 0.37 |
| ^*^CO + H^+^ + e^-^ → ^*^COH | 1.41 |
| ^*^CHO + H^+^ + e^-^ → ^*^CH_2_O | –0.57 |
| ^*^CHO + H^+^ + e^-^ → ^*^CHOH | 0.53 |
| ^*^CH_2_O + H^+^ + e^-^ → ^*^CH_3_O | –0.61 |
| ^*^CH_2_O + H^+^ + e^-^ →^*^CH_2_OH | –0.03 |
| ^*^CH_3_O + H^+^ + e^-^ → CH_4_ + ^*^O | –1.18 |
| ^*^CH_3_O + H^+^ + e^-^ → ^*^CH_3_OH | 0.21 |
| ^*^O + H^+^ + e^-^ → ^*^OH | 0.09 |
| ^*^OH + H^+^ + e^-^ → ^*^H_2_O | 0.13 |
| ^*^H_2_O → H_2_O + ^*^ | 0.69 |

| Zn/g−CN | *∆G* |
| --- | --- |
| ^*^ + CO → ^*^CO | –0.48 |
| ^*^CO + H^+^ + e^-^ →^*^CHO | 0.44 |
| ^*^CO + H^+^ + e^-^ → ^*^COH | 2.77 |
| ^*^CHO + H^+^ + e^-^ → ^*^CH_2_O | 0.48 |
| ^*^CHO + H^+^ + e^-^ → ^*^CHOH | 0.67 |
| ^*^CH_2_O + H^+^ + e^-^ → ^*^CH_3_O | –1.27 |
| ^*^CH_2_O + H^+^ + e^-^ →^*^CH_2_OH | –0.83 |
| ^*^CH_3_O + H^+^ + e^-^ → ^*^CH_3_OH | –0.73 |
| ^*^CH_3_O + H^+^ + e^-^ → CH_4_ + ^*^O | 1.19 |
| ^*^CH_3_OH → CH_3_OH + ^*^ | 1.03 |

**Table S5.** The computed free energy changes (*ΔG*, eV) of all potential elementary steps from COER to C_2_ products on V/g−CN and Zn/g−CN catalysts. The most favorable steps were marked in red.

| V/g−CN | *∆G* |
| --- | --- |
| ^*^CHO + CO → ^*^CHO–CO | –0.07 |
| ^*^CHO–CO + H^+^ + e^-^ →^*^CHO–CHO | –0.53 |
| ^*^CHO–CO + H^+^ + e^-^→ ^*^CH_2_O–CO | –0.34 |
| ^*^CHO–CO + H^+^ + e^-^ → ^*^CHO–COH | 0.03 |
| ^*^CHO–CO + H^+^ + e^-^ → ^*^CHOH–CO | 0.43 |
| ^*^CHO–CHO + H^+^ + e^-^ → ^*^CHO–CH_2_O | –0.54 |
| ^*^CHO–CHO + H^+^ + e^-^ → ^*^CH_2_O–CHO | –0.29 |
| ^*^CHO–CHO + H^+^ + e^-^ → ^*^CHOH–CHO | –0.10 |
| ^*^CHO–CHO + H^+^ + e^-^ → ^*^CHO–CHOH | –0.20 |
| ^*^CHO–CH_2_O + H^+^ + e^-^ → ^*^CHO–CH_3_O | –0.52 |
| ^*^CHO–CH_2_O + H^+^ + e^-^ →^*^CH_2_O–CH_2_O | –0.39 |
| ^*^CHO–CH_2_O + H^+^ + e^-^ → ^*^CHO–CH_2_OH | 0.69 |
| ^*^CHO–CH_2_O + H^+^ + e^-^ →^*^CHOH–CH_2_O | 1.21 |
| ^*^CHO–CH_3_O + H^+^ + e^-^ → ^*^CH_2_O–CH_3_O | –0.82 |
| ^*^CHO–CH_3_O + H^+^ + e^-^ → ^*^CHOH–CH_3_O | 0.23 |
| ^*^CHO–CH_3_O + H^+^ + e^-^ → ^*^CHO–CH_3_OH | 0.02 |
| ^*^CH_2_O–CH_3_O + H^+^ + e^-^ →^*^CH_2_OH–CH_3_O | –0.12 |
| ^*^CH_2_O–CH_3_O + H^+^ + e^-^ →^*^CH_2_O–CH_3_OH | 0.29 |
| ^*^CH_2_OH–CH_3_O → CH_3_CH_2_OH + ^*^O | 0.12 |
| ^*^CH_2_OH–CH_3_O + H^+^ + e^-^ → ^*^CH_2_OH–CH_3_OH | 0.16 |
| ^*^O + H^+^ + e^-^ → ^*^OH | 0.09 |
| ^*^OH + H^+^ + e^-^ → ^*^H_2_O | 0.13 |
| ^*^H_2_O → H_2_O + ^*^ | 0.69 |

| Zn/g−CN | *∆G* |
| --- | --- |
| ^*^CHO + CO → ^*^CHO–CO | 0.23 |
| ^*^CHO–CO + H^+^ + e^-^ →^*^CHO–CHO | –0.11 |
| ^*^CHO–CO + H^+^ + e^-^ → ^*^CH_2_O–CO | 0.47 |
| ^*^CHO–CO + H^+^ + e^-^ → ^*^CHO–COH | 1.17 |
| ^*^CHO–CO + H^+^ + e^-^ → ^*^CHOH–CO | –0.04 |
| ^*^CHO–CHO + H^+^ + e^-^ → ^*^CHO–CH_2_O | –1.05 |
| ^*^CHO–CHO + H^+^ + e^-^ → ^*^CH_2_O–CHO | –0.74 |
| ^*^CHO–CHO + H^+^ + e^-^ → ^*^CHOH–CHO | –0.70 |
| ^*^CHO–CHO + H^+^ + e^-^ → ^*^CHO–CHOH | –0.45 |
| ^*^CHO–CH_2_O + H^+^ + e^-^ → ^*^CHO–CH_2_OH | 0.47 |
| ^*^CHO–CH_2_O + H^+^ + e^-^ →^*^CH_2_O–CH_2_O | 0.89 |
| ^*^CHO–CH_2_O + H^+^ + e^-^ → ^*^CHO–CH_3_O | 1.81 |
| ^*^CHO–CH_2_O + H^+^ + e^-^ →^*^CHOH–CH_2_O | 1.10 |
| ^*^CHO–CH_2_OH + H^+^ + e^-^ → ^*^CHO–CH_2_ + H_2_O | –1.08 |
| ^*^CHO–CH_2_OH + H^+^ + e^-^ → ^*^CHOH–CH_2_OH | –0.15 |
| ^*^CHO–CH_2_OH + H^+^ + e^-^ → ^*^CH_2_O–CH_2_OH | –0.59 |
| ^*^CHO–CH_2_ + H^+^ + e^-^ →^*^CHO–CH_3_ | –0.21 |
| ^*^CHO–CH_2_ + H^+^ + e^-^ →^*^CH_2_O–CH_2_ | 1.24 |
| ^*^CHO–CH_2_ + H^+^ + e^-^ →^*^CHOH–CH_2_ | 0.06 |
| ^*^CHO–CH_3_ + H^+^ + e^-^ → ^*^CH_2_O–CH_3_ | –0.21 |
| ^*^CHO–CH_3_ + H^+^ + e^-^ → ^*^CHOH–CH_3_ | 0.19 |
| ^*^CH_2_O–CH_3_ + H^+^ + e^-^ → ^*^CH_2_OH–CH_3_ | –0.68 |
| ^*^CH_2_OH–CH_3_ → CH_3_CH_2_OH + ^*^ | 0.79 |

**Table S6.** Elastic constants (*C_11_*, *C_22_*, *C_12_*, and *C_66_*, in N m^-1^) and Young’s modulus (*Y_x_*, *Y_y_*: the Y value along the x, y direction, in N m^-1^)

| System | *C_11_* | *C_22_* | *C_12_* | *C_66_* | *Y_x_* | *Y_y_* |
| --- | --- | --- | --- | --- | --- | --- |
| V/g−CN | 124.09 | 131.10 | 27.60 | 54.24 | 118.28 | 124.96 |
| Zn/g−CN | 121.85 | 125.83 | 40.37 | 49.29 | 108.89 | 112.45 |

**
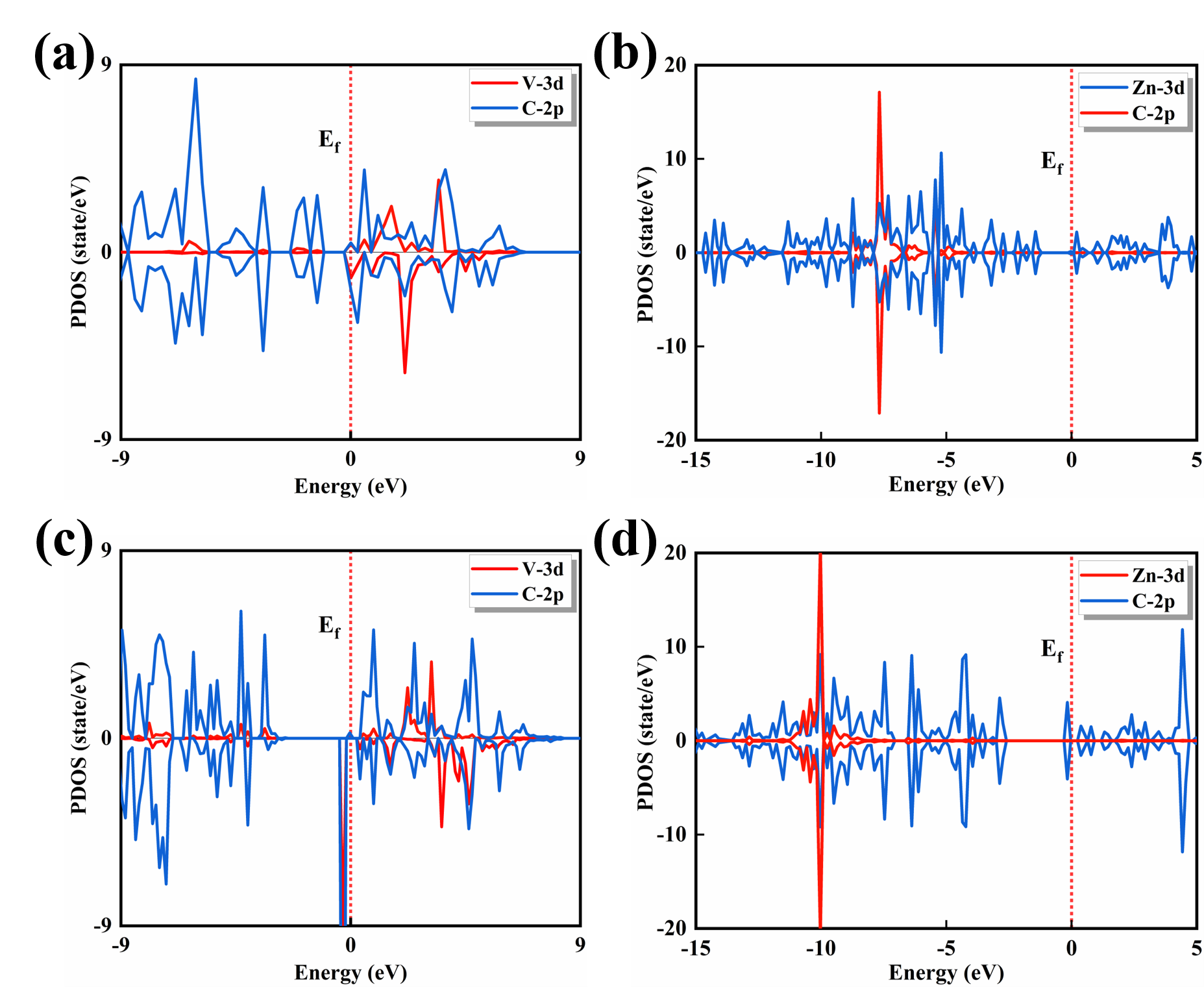
**

**Fig. S1.** The projected density of states (PDOS) for CO adsorption is presented for (a) V/g−CN and (b) Zn/g−CN catalysts using the PBE functional, as well as for (c) V/g−CN and (d) Zn/g−CN catalysts using the HSE06 functional.





**Fig. S2. The computed** phonon spectrum of pristine g-CN monolayer.

**
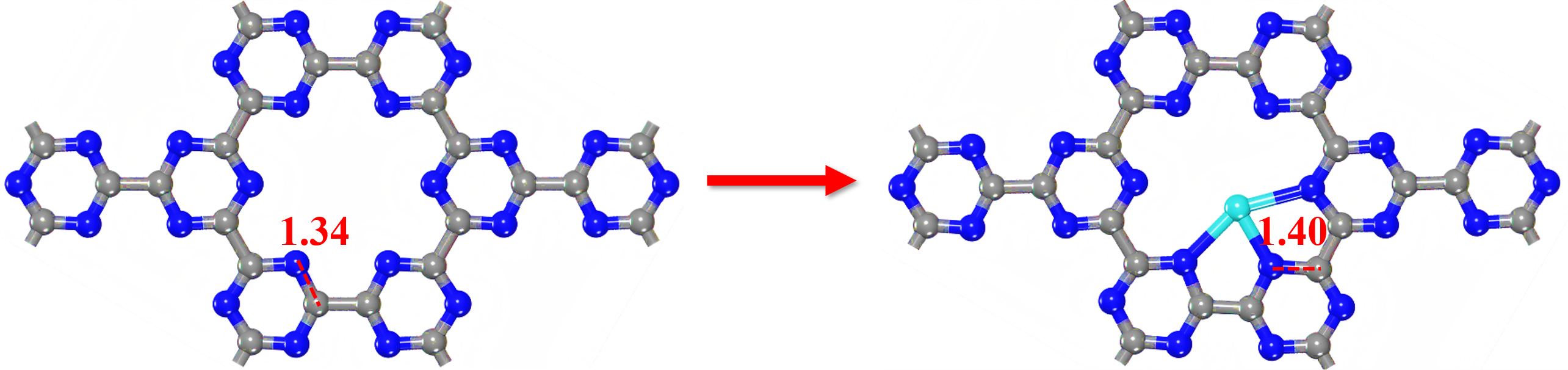
**

**Fig. S3.** The variation of N–C bond lengths between metal-coordinated nitrogen atoms and their adjacent carbon atoms.

**
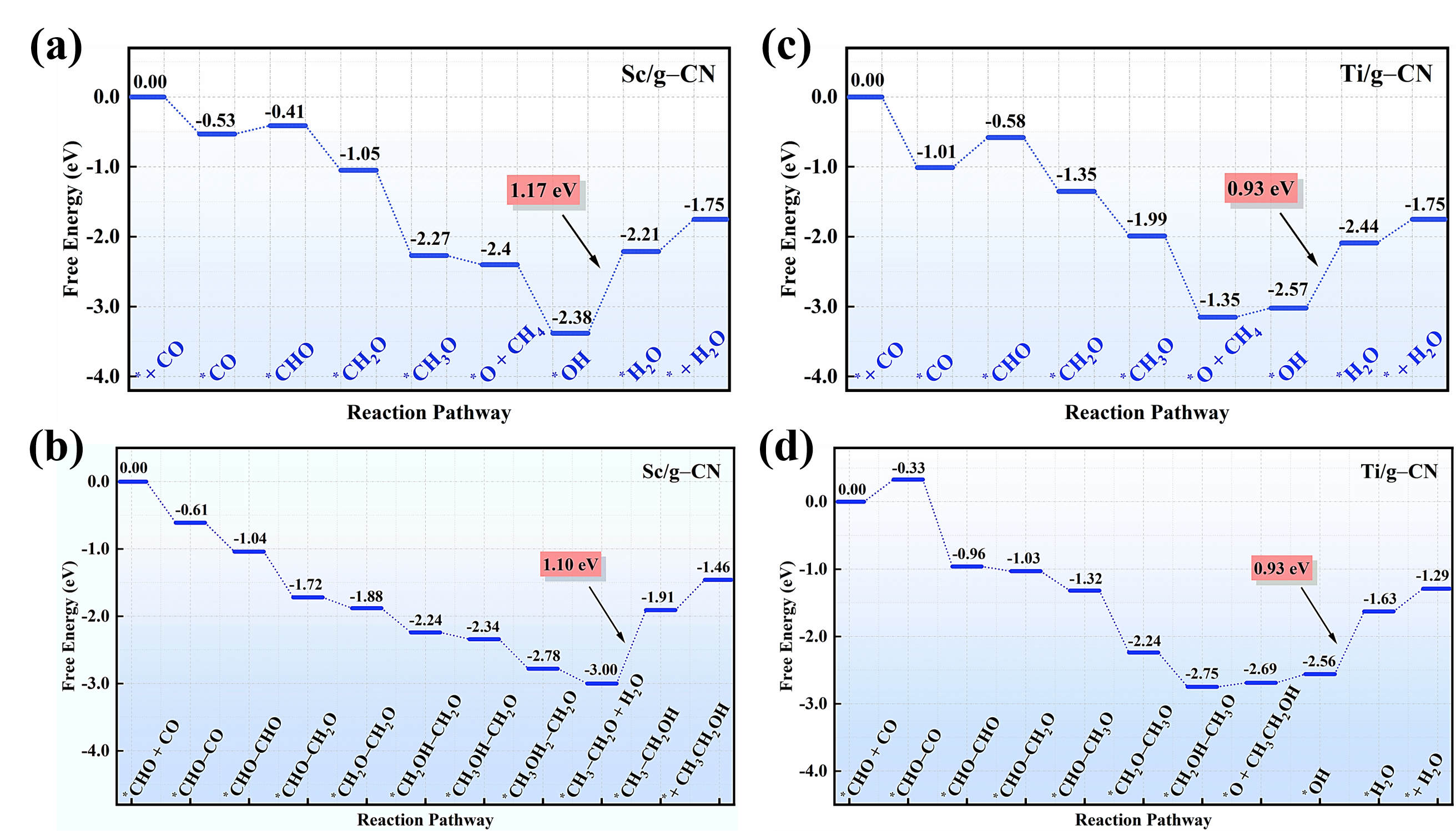
**

**Fig. S4.** The most favorable free energy profiles for COER to C_1_ products on the (a) Sc/g−CN and (b) Ti/g−CN catalysts, and to C_2_H_5_OH production on (c) Sc/g−CN and (d) Ti/g−CN catalysts.

**
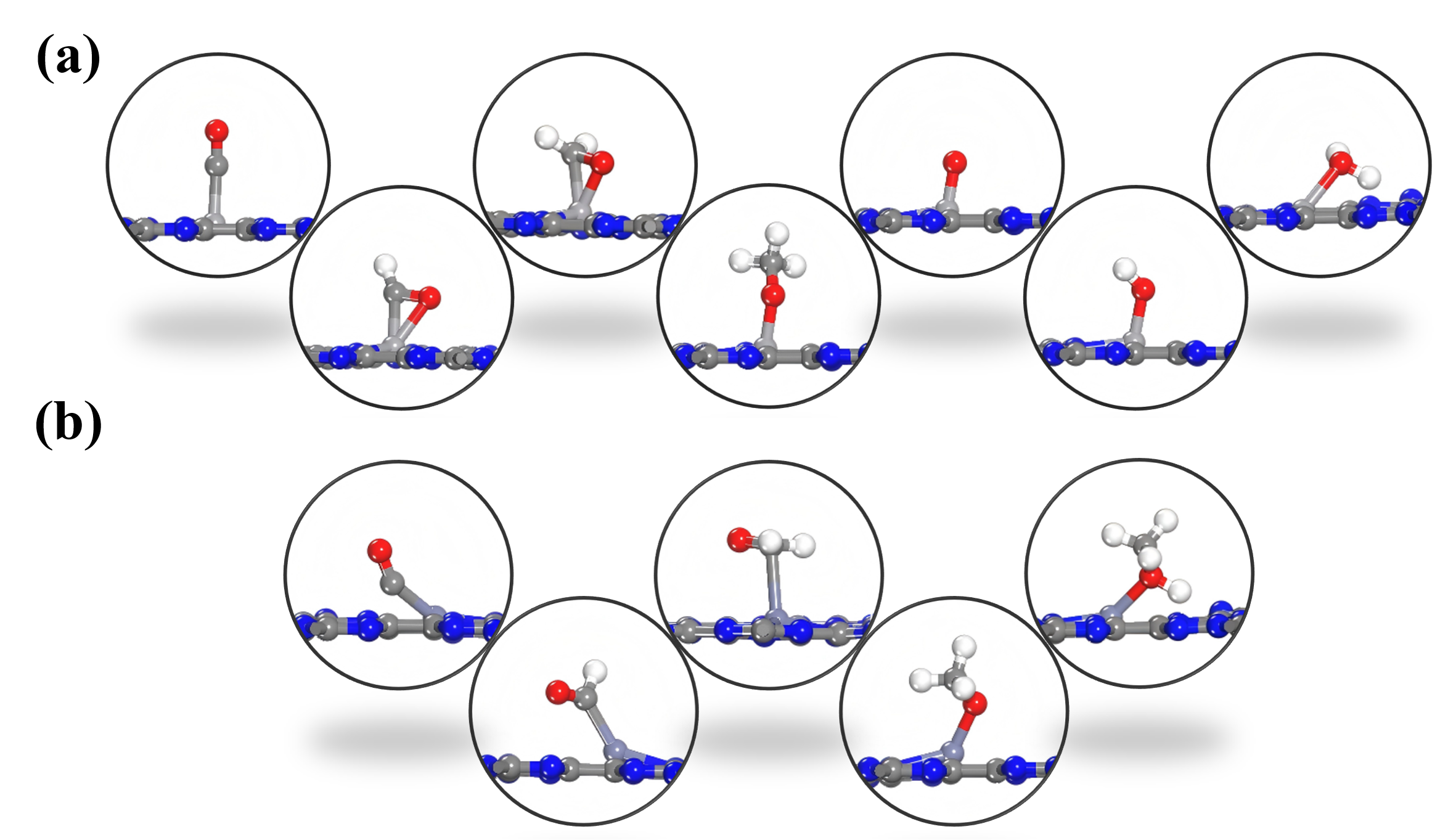
**

**Fig. S5.** The corresponding intermediates involved in the lowest energy path of the COER to produce C_1_ products on (a) V/g−CN and (b) Zn/g−CN catalysts.


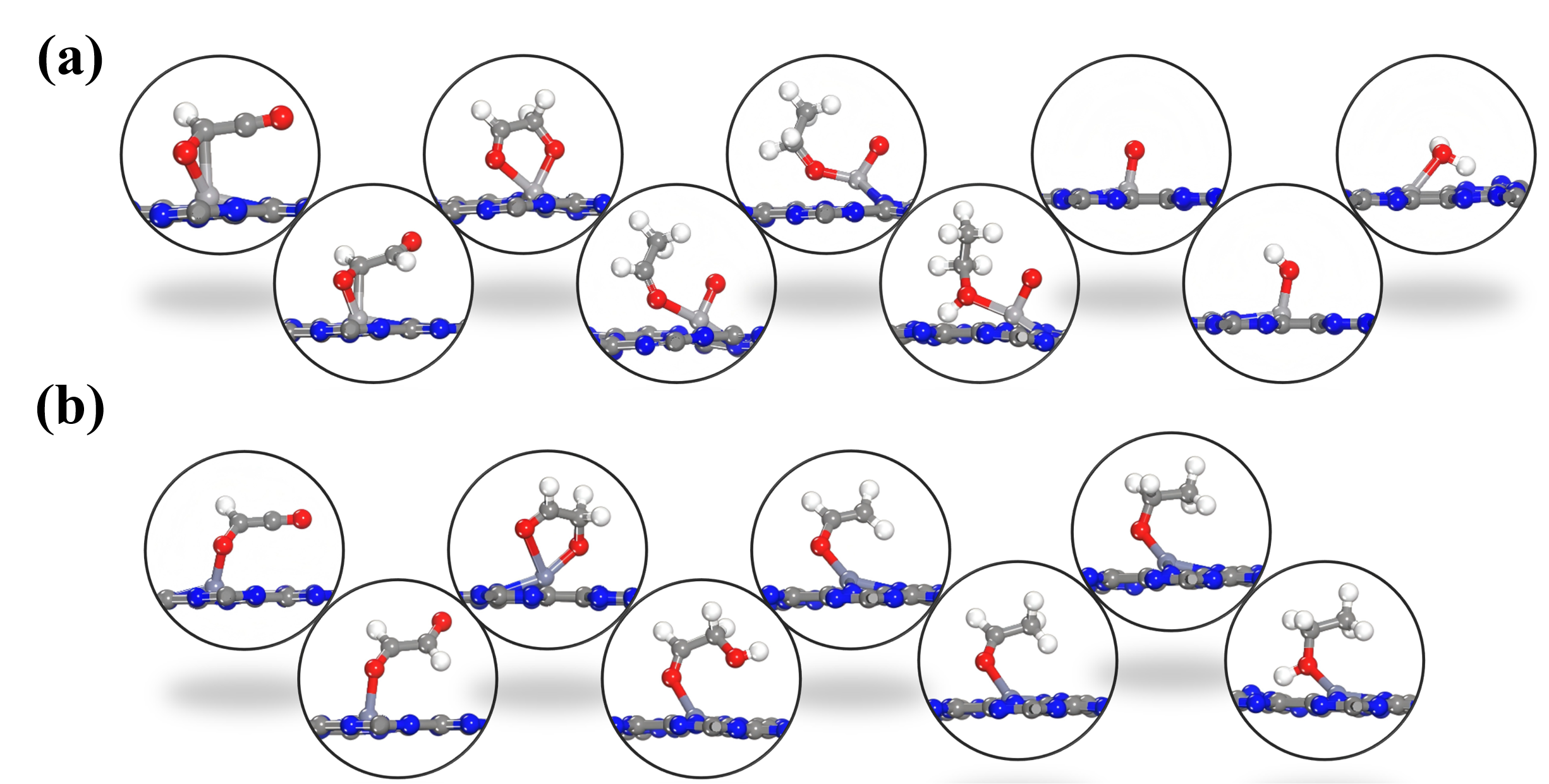


**Fig. S6.** The corresponding intermediates involved in the lowest energy path of the COER to produce C_2_ products on (a) V/g−CN and (b) Zn/g−CN catalysts.





**Fig. S7.** The hydrogen evolution reaction on V/g−CN and Zn/g−CN catalysts.


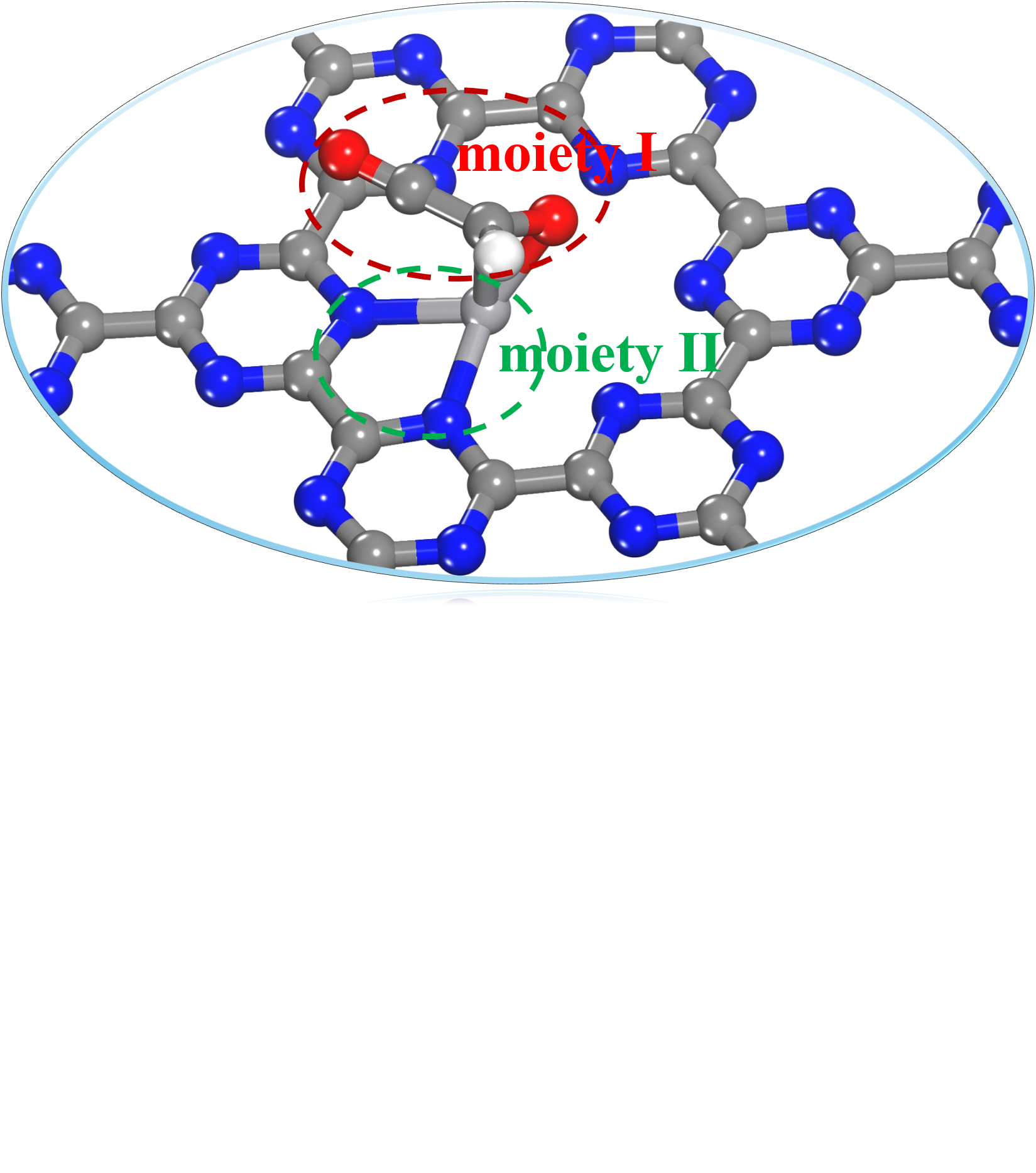


**Fig. S8.** Three adsorbed ^*^C_x_H_y_O_z_ intermediates on V/g−CN, which was divided into three moieties: moiety Ⅰ: absorbed molecules; moiety Ⅱ: TM−N_2_; and moiety Ⅲ: g−CN substrate).


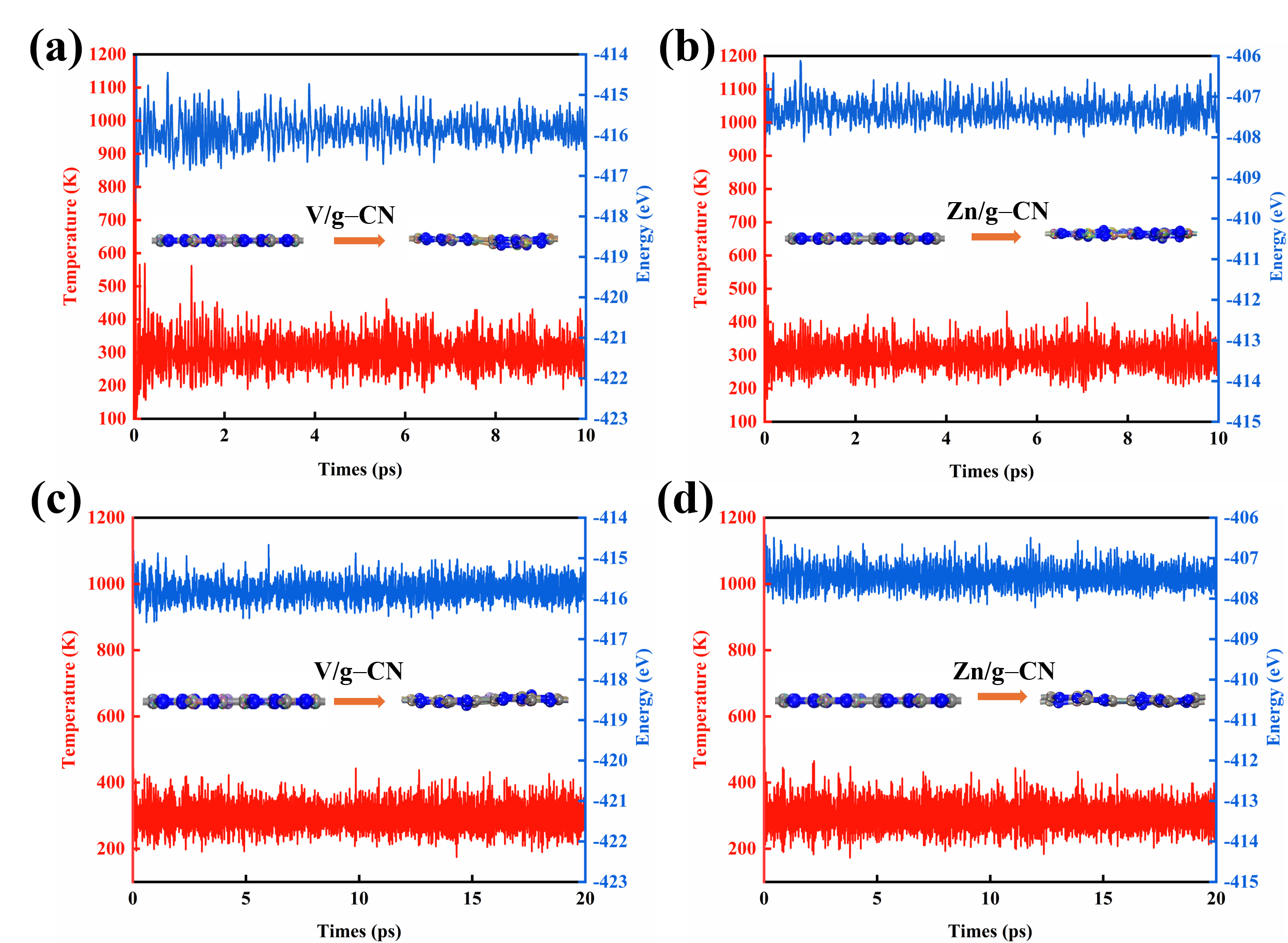


**Fig. S9.** Temperature and energy changes over time in the AIMD simulation of (a) (c) V/g−CN and (b) (d) Zn/g−CN catalysts. The simulations were performed for 10 ps and 20 ps at 300 K, respectively.


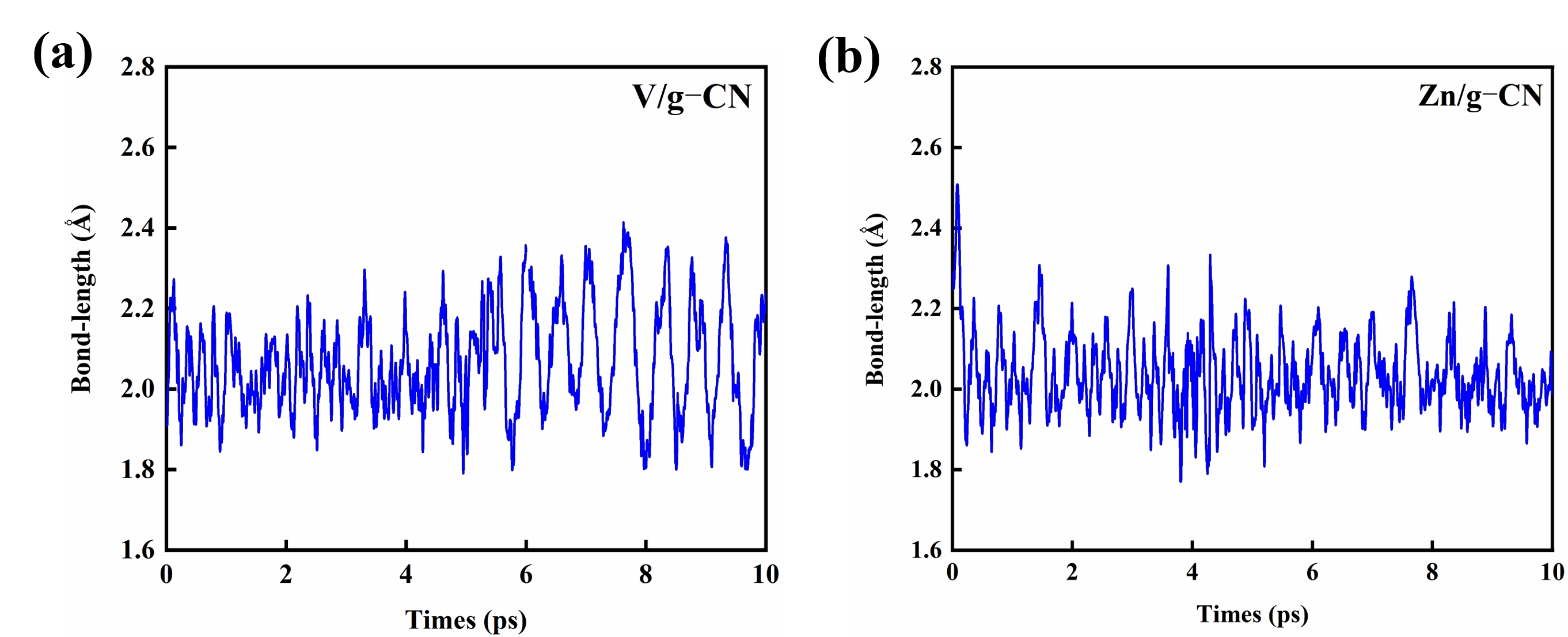


**Fig. S10.** TM–N bond length over time in the AIMD simulation of (a) V/g−CN and (b) Zn/g−CN catalysts. The simulations were performed for 10 ps at 300 K.

The detail structural information for the V and Zn reaction pathways including reactant, transition states and intermediates/products

V/g−CN

1.0

14.2230997086 0.0000000000 0.0000000000

-7.1115498543 12.3175656682 0.0000000000

0.0000000000 0.0000000000 20.0000000000

C N V

24 24 1

Direct

0.227460003 0.439950012 0.500000000

0.063669998 0.282990012 0.500000000

0.219359995 0.275849996 0.500000000

0.276730003 0.054750002 0.500000000

0.437229992 0.212929990 0.500000000

0.274529979 0.212969999 0.500000000

0.711080030 0.439900035 0.500000000

0.555289987 0.275880018 0.500000000

0.718190037 0.283100012 0.500000000

0.782549975 0.066299997 0.500000000

0.941760067 0.224990005 0.500000000

0.782149936 0.225030014 0.500000000

0.216919998 0.935180011 0.500000000

0.058030021 0.776000054 0.500000000

0.216570014 0.776059980 0.500000000

0.285659997 0.560769974 0.500000000

0.441339979 0.721750037 0.500000000

0.280039995 0.716860019 0.500000000

0.721170009 0.943670009 0.500000000

0.562650015 0.784780005 0.500000000

0.720619965 0.784749965 0.500000000

0.773350019 0.560850030 0.500000000

0.935350013 0.716819991 0.500000000

0.778980053 0.721830016 0.500000000

0.387169995 0.275580000 0.500000000

0.218670005 0.106440006 0.500000000

0.386449976 0.106399997 0.500000000

0.111629999 0.222389994 0.500000000

0.287340001 0.387800002 0.500000000

0.119940006 0.392480008 0.500000000

0.891530005 0.284179998 0.500000000

0.723349965 0.115720000 0.500000000

0.891140017 0.115649999 0.500000000

0.609660019 0.222430002 0.500000000

0.771340047 0.392630017 0.500000000

0.599170012 0.387700009 0.500000000

0.389189999 0.777800006 0.500000000

0.224490012 0.608299981 0.500000000

0.394670007 0.612420018 0.500000000

0.107299981 0.716019969 0.500000000

0.275489973 0.884259997 0.500000000

0.107390014 0.884240022 0.500000000

0.887170050 0.777809993 0.500000000

0.716099997 0.612500036 0.500000000

0.882090046 0.608270018 0.500000000

0.611969977 0.725429996 0.500000000

0.779720024 0.893240011 0.500000000

0.612150033 0.893280039 0.500000000

0.466009991 0.434929999 0.500000000

V-CO

1.0

14.2230997086 0.0000000000 0.0000000000

-7.1115498543 12.3175656682 0.0000000000

0.0000000000 0.0000000000 20.0000000000

C N V O

25 24 1 1

Direct

0.227780008 0.439869994 0.500309992

0.063809999 0.283010006 0.500000000

0.219360004 0.275620028 0.499959993

0.276730002 0.054500000 0.499489975

0.437180009 0.212630011 0.500029993

0.274459980 0.212680007 0.500090027

0.710919978 0.439910022 0.500139999

0.555170018 0.275580000 0.499819994

0.718170014 0.283090005 0.499989986

0.782620034 0.066419999 0.500050020

0.941820052 0.225140014 0.500010014

0.782229978 0.225150002 0.500000000

0.216769984 0.934689995 0.499879980

0.057840020 0.775600005 0.500000000

0.216410016 0.775530013 0.500090027

0.286200015 0.560750037 0.500070000

0.441500028 0.721760025 0.499959993

0.280010010 0.716509985 0.500169992

0.721189980 0.943809992 0.500050020

0.562730027 0.784979991 0.499970007

0.720690056 0.785000044 0.499989986

0.773060040 0.560830016 0.500000000

0.935180020 0.716650045 0.500090027

0.778900058 0.721920060 0.499980021

0.474400048 0.441450023 0.607219982

0.387009996 0.275040007 0.501559973

0.218699995 0.106160003 0.499170017

0.386380003 0.106099999 0.499130011

0.111580008 0.222230016 0.499819994

0.287250013 0.387360002 0.500220013

0.120300010 0.392510010 0.500240040

0.891630052 0.284339995 0.500020027

0.723449967 0.115850004 0.500010014

0.891209999 0.115820002 0.500060034

0.609629966 0.222259999 0.499860001

0.771100004 0.392630017 0.500190020

0.598919971 0.387310025 0.499900007

0.389140030 0.777570057 0.500360012

0.224760003 0.607940037 0.500209999

0.395139968 0.612420018 0.499590015

0.107140007 0.715660026 0.499889994

0.275320012 0.883840050 0.500090027

0.107350006 0.883920028 0.500040007

0.887070051 0.777720027 0.500320005

0.715830046 0.612520011 0.499639988

0.881729959 0.608090007 0.500080013

0.612070035 0.725679998 0.499889994

0.779790015 0.893450062 0.500090027

0.612170035 0.893470038 0.500050020

0.466609993 0.434209995 0.509239960

0.475049979 0.435309996 0.665130043

V-CHO

1.0

14.2230997086 0.0000000000 0.0000000000

-7.1115498543 12.3175656682 0.0000000000

0.0000000000 0.0000000000 20.0000000000

C N V O H

25 24 1 1 1

Direct

0.210460009 0.429080002 0.504430008

0.054040002 0.267219996 0.503099966

0.214300016 0.268020018 0.504059982

0.283919990 0.056999999 0.500969982

0.440609998 0.220400005 0.500540018

0.277369996 0.212689995 0.502290010

0.723879988 0.432430019 0.498180008

0.562200016 0.278470022 0.499760008

0.716680015 0.270320012 0.503690004

0.775080024 0.047460002 0.504349995

0.934270024 0.206209999 0.503570032

0.775010016 0.205860004 0.504290009

0.225909980 0.934509984 0.500379992

0.067430000 0.775690049 0.500100040

0.226380034 0.775410006 0.499100018

0.272450006 0.546430017 0.502180004

0.439020006 0.701799981 0.501399994

0.283050029 0.710680041 0.499800014

0.715590017 0.924780042 0.504339981

0.559490042 0.763039985 0.502510023

0.720759976 0.768370003 0.504390001

0.786949985 0.554080004 0.498920012

0.944700000 0.713429984 0.500609970

0.785029987 0.711120041 0.503210020

0.470869989 0.517679996 0.612330008

0.388120013 0.278940005 0.501280022

0.223100015 0.104290002 0.502330017

0.393310016 0.112630001 0.500150013

0.107850000 0.211130000 0.502180004

0.275260011 0.380949998 0.507859993

0.104040005 0.376720019 0.502419996

0.883189942 0.264570008 0.503889990

0.715019996 0.096699999 0.504580021

0.883400012 0.096730000 0.503849983

0.608120038 0.216030005 0.504660034

0.778830001 0.379300016 0.502430010

0.614989983 0.386579995 0.494280005

0.392489996 0.762390012 0.500139999

0.220910012 0.602129991 0.501230001

0.384870026 0.589220015 0.501980019

0.117020000 0.716949986 0.498950005

0.285470048 0.884990027 0.499460030

0.116760025 0.884380004 0.501079988

0.893580024 0.771170040 0.503620052

0.726940003 0.602680049 0.501130009

0.895430015 0.604489988 0.497970009

0.611090041 0.705380064 0.503299999

0.776459962 0.876429960 0.505680037

0.607300017 0.871819985 0.502430010

0.433089979 0.471789983 0.520659971

0.514960015 0.461030031 0.604130030

0.477830000 0.560440032 0.659870052

V-CH_2_O

1.0

14.2230997086 0.0000000000 0.0000000000

-7.1115498543 12.3175656682 0.0000000000

0.0000000000 0.0000000000 20.0000000000

C N V O H

25 24 1 1 2

Direct

0.213719991 0.431039989 0.506120014

0.058200000 0.268070014 0.505320024

0.220139996 0.271200012 0.503539991

0.284500010 0.056559999 0.499970007

0.442710024 0.217730002 0.498719978

0.281879994 0.214160004 0.500859976

0.725079989 0.430499995 0.498829985

0.564310021 0.275570012 0.499100018

0.719859999 0.269360013 0.502709961

0.778749981 0.047210000 0.504729986

0.938269948 0.205960017 0.505500031

0.778980001 0.205760011 0.504040003

0.224759982 0.934069984 0.499630022

0.066429992 0.775229996 0.499529982

0.225330022 0.775320040 0.500839996

0.273629991 0.548200005 0.504819965

0.439339992 0.704170005 0.501689959

0.282849997 0.711500038 0.501460028

0.717790022 0.924360017 0.504379988

0.559979982 0.764119972 0.501309967

0.719479962 0.765900025 0.501329994

0.786769964 0.552049988 0.499030018

0.943790021 0.712299982 0.499240017

0.783270037 0.708289964 0.500670004

0.458479983 0.525889991 0.618989992

0.392019991 0.277450001 0.498040009

0.225379997 0.105520007 0.501669979

0.393909991 0.109779997 0.499380016

0.113760000 0.213640006 0.503350019

0.280669994 0.384650010 0.505940008

0.107189997 0.377369993 0.506120014

0.887040040 0.264330013 0.505119991

0.718950000 0.096560007 0.503590012

0.887209983 0.096290000 0.505649996

0.611099985 0.213870013 0.502419996

0.781149995 0.378360013 0.501909971

0.616250025 0.383950020 0.496180010

0.392670032 0.764490058 0.499959993

0.221290020 0.603290034 0.503419971

0.385769989 0.592549980 0.504859972

0.116130021 0.716610017 0.500839996

0.284279979 0.884599965 0.500410032

0.115680024 0.884040036 0.498659992

0.891820023 0.769190000 0.500269985

0.725740003 0.599810022 0.500750017

0.895220021 0.603330023 0.497919989

0.609550001 0.704429995 0.498559999

0.776489963 0.873930019 0.504260015

0.609179978 0.872629994 0.504129982

0.437200011 0.476520004 0.522830009

0.457159994 0.430620041 0.604170036

0.387419989 0.517080038 0.646250010

0.537489990 0.594929991 0.633660030

V-CH_3_O

1.0

14.2230997086 0.0000000000 0.0000000000

-7.1115498543 12.3175656682 0.0000000000

0.0000000000 0.0000000000 20.0000000000

C N V O H

25 24 1 1 3

Direct

0.212599999 0.428250017 0.503139973

0.057090013 0.265660001 0.502979994

0.218579997 0.268390007 0.500279999

0.283829968 0.053949999 0.499889994

0.441819999 0.215320008 0.499259996

0.280630008 0.211440005 0.499679995

0.724219958 0.428649989 0.498719978

0.563430042 0.273490018 0.499300003

0.718930025 0.267369986 0.502300024

0.777879993 0.045209999 0.504050016

0.937080027 0.203970009 0.503570032

0.777919994 0.203710000 0.503480005

0.224589997 0.931350004 0.500320005

0.066670015 0.772430037 0.500880003

0.225909972 0.772779993 0.502390003

0.273270027 0.545590006 0.504310036

0.440370033 0.702580027 0.503269958

0.283300029 0.709620030 0.503119993

0.717549955 0.922619992 0.503740025

0.559930057 0.762140010 0.502340031

0.720200063 0.764800064 0.500370026

0.786539957 0.550289987 0.498499966

0.944079968 0.709950011 0.500370026

0.783949984 0.707019980 0.499510002

0.453780020 0.538810032 0.655529976

0.391059984 0.274890017 0.499289989

0.224460008 0.102710002 0.499769974

0.393310014 0.107410002 0.499650002

0.111859994 0.210679992 0.501420021

0.278759995 0.381470016 0.500469971

0.106069991 0.375039997 0.504320049

0.886100007 0.262479987 0.503319979

0.718060053 0.094619995 0.503999996

0.886340053 0.094480003 0.503859997

0.610210047 0.211800007 0.502059984

0.780170106 0.376340022 0.501339960

0.615259993 0.381960032 0.496880007

0.392860009 0.761969987 0.502579975

0.221599982 0.600589990 0.503520012

0.386410013 0.589350010 0.505130005

0.116430008 0.714010044 0.502530003

0.284449991 0.882449980 0.501410007

0.115490009 0.881239999 0.499620008

0.892500046 0.767310070 0.500800037

0.726059981 0.598570000 0.497779989

0.894959984 0.600920048 0.499679995

0.610210057 0.702820041 0.499340010

0.777119982 0.872750001 0.502309990

0.609399979 0.870980013 0.504800034

0.436970003 0.478720005 0.513890028

0.458460003 0.476469988 0.601679993

0.372540021 0.530600037 0.658330011

0.515120038 0.625450039 0.649670029

0.469400001 0.508119999 0.702350044

V-O

1.0

14.2230997086 0.0000000000 0.0000000000

-7.1115498543 12.3175656682 0.0000000000

0.0000000000 0.0000000000 20.0000000000

C N V O

24 24 1 1

Direct

0.212270007 0.426930017 0.503109980

0.054720002 0.265520019 0.502489996

0.213970022 0.264640018 0.501460028

0.284619997 0.054770001 0.500150013

0.441440007 0.218940003 0.501030016

0.277020018 0.210020011 0.500790024

0.724319968 0.430990011 0.499000025

0.562650031 0.276710002 0.500810051

0.717299979 0.268710001 0.502379990

0.775919947 0.046190002 0.503870010

0.935050012 0.205030001 0.502900028

0.775829974 0.204559999 0.503099966

0.227269984 0.932600013 0.500400019

0.068500014 0.773410069 0.500829983

0.227339993 0.773219993 0.501779985

0.274670014 0.544380025 0.503269958

0.440380012 0.699620033 0.503030014

0.284209969 0.708190010 0.502340031

0.716929948 0.923439989 0.503940010

0.561390038 0.761520076 0.502540016

0.722440011 0.767290017 0.499860001

0.787760046 0.552489988 0.498390007

0.945850022 0.711450022 0.500339985

0.786599990 0.709969986 0.498820019

0.388489977 0.277740011 0.501550007

0.223690012 0.101960006 0.500040007

0.394320008 0.111560002 0.500329971

0.107610002 0.208470004 0.501299953

0.275850008 0.377559991 0.502260017

0.105880010 0.375330027 0.503090000

0.883989999 0.263449993 0.502640009

0.715789933 0.095310007 0.503819990

0.884190010 0.095520000 0.503400040

0.608720021 0.214279992 0.503310013

0.779259944 0.377779991 0.501009989

0.615069945 0.385220005 0.497720003

0.394000014 0.760420037 0.502139997

0.222470013 0.599960030 0.502890015

0.386539996 0.587710017 0.503600025

0.118120013 0.714530023 0.501719999

0.286699976 0.882689995 0.501169968

0.117969975 0.882059996 0.500040007

0.895070029 0.769660041 0.500440025

0.728129965 0.601469991 0.496950006

0.896249997 0.602439996 0.500000000

0.612949995 0.704389966 0.498540020

0.777950009 0.875410055 0.502200031

0.608439936 0.869989993 0.505450010

0.433279975 0.459100007 0.518369961

0.455260007 0.469639998 0.597819996

V-OH

1.0

14.2230997086 0.0000000000 0.0000000000

-7.1115498543 12.3175656682 0.0000000000

0.0000000000 0.0000000000 20.0000000000

C N V O H

24 24 1 1 1

Direct

0.214009996 0.429080002 0.506579971

0.059090002 0.266100001 0.503299999

0.221640010 0.270460014 0.503310013

0.282970018 0.053300001 0.500259972

0.442110000 0.213129995 0.501230001

0.282559993 0.212190011 0.501169968

0.723660025 0.427210020 0.500559998

0.563730004 0.271230014 0.501490021

0.720029992 0.266699998 0.503220034

0.779440025 0.045040001 0.502680016

0.938839978 0.203810012 0.503260040

0.779720025 0.203669991 0.503590012

0.222450003 0.930710018 0.500029993

0.064259999 0.771910058 0.500390005

0.223270000 0.772029987 0.502789974

0.272740000 0.546440005 0.505739975

0.439189983 0.704929998 0.503409958

0.281080002 0.709580002 0.503719997

0.717630064 0.922400030 0.502290010

0.558640016 0.763390019 0.502960014

0.717360025 0.762969994 0.501470041

0.784660002 0.548890008 0.499940014

0.941700009 0.709130014 0.500220013

0.781020012 0.705050005 0.500289965

0.392560008 0.273730013 0.500850010

0.224820002 0.103349998 0.500479984

0.392430023 0.105170002 0.501200008

0.114930003 0.212270009 0.501339960

0.281540025 0.383450016 0.508099985

0.107479995 0.375250009 0.504600000

0.887930015 0.262369987 0.503960037

0.719919958 0.094700003 0.502990007

0.888019981 0.094260003 0.502619982

0.611199973 0.210209990 0.503419971

0.780530010 0.375669996 0.502200031

0.614810021 0.379710015 0.499609995

0.390860026 0.763360056 0.502900028

0.220100026 0.600470022 0.505360031

0.385700003 0.591820027 0.504580021

0.113779986 0.713359993 0.502699947

0.281920007 0.881610008 0.501850033

0.113319992 0.880900031 0.498589993

0.889559982 0.765860075 0.500460052

0.723479970 0.596520008 0.499529982

0.893079984 0.600149990 0.500309992

0.607360019 0.702220006 0.502619982

0.775569978 0.870960037 0.501359987

0.609449987 0.872189994 0.502729988

0.440510015 0.487950021 0.519589996

0.467479984 0.494859990 0.608059978

0.417020001 0.447580024 0.642119980

V-H_2_O

1.0

14.2230997086 0.0000000000 0.0000000000

-7.1115498543 12.3175656682 0.0000000000

0.0000000000 0.0000000000 20.0000000000

C N V O H

24 24 1 1 2

Direct

0.210280020 0.421330022 0.499399996

0.048790005 0.263260014 0.497970009

0.203739996 0.255019998 0.499709988

0.275460004 0.044390003 0.498499966

0.431999998 0.208750007 0.499779987

0.264570005 0.197929994 0.500240040

0.711799996 0.427480036 0.512220001

0.551960007 0.270289991 0.501500034

0.709640040 0.267230003 0.507509995

0.770060032 0.046720003 0.504209995

0.928990014 0.205489995 0.499480009

0.769900028 0.205080016 0.505310011

0.218260014 0.923829973 0.499930000

0.059209987 0.764869977 0.504329967

0.217509996 0.764069956 0.501820040

0.273560000 0.540139981 0.501100016

0.435230016 0.697550027 0.506099987

0.277129996 0.701259949 0.502399969

0.712090038 0.924460049 0.506789970

0.556770014 0.761979974 0.508990002

0.719109982 0.769949994 0.516919994

0.777039970 0.548800002 0.516669989

0.937100009 0.705829974 0.507560015

0.781469991 0.710839999 0.519490004

0.377430011 0.266779996 0.502510023

0.213199993 0.091339998 0.498650026

0.385129993 0.101790003 0.497630024

0.097339999 0.202120003 0.498409986

0.269499996 0.367950017 0.500850010

0.103600008 0.373020007 0.497870016

0.878200054 0.264250014 0.501699972

0.710060027 0.095770002 0.507179976

0.878010001 0.096070000 0.500010014

0.601689986 0.211350000 0.501770020

0.768270018 0.375990027 0.515339947

0.602339978 0.380470008 0.503950024

0.386599989 0.756710037 0.505680037

0.218049991 0.593029970 0.500010014

0.384530022 0.586359976 0.504100037

0.108210025 0.704860007 0.504250002

0.276959994 0.872850033 0.499270010

0.108860013 0.873160038 0.502589989

0.888189970 0.766129974 0.509570026

0.722339996 0.601860014 0.528620005

0.883789999 0.596589999 0.506250000

0.609810006 0.706860022 0.517019987

0.774259996 0.877910073 0.512889957

0.603330008 0.870010046 0.502960014

0.428460010 0.428830000 0.511049986

0.532000014 0.505620020 0.590569973

0.607779986 0.544420014 0.566730022

0.523320052 0.566030039 0.605799961

V-CHO-CO

1.0

14.2230997086 0.0000000000 0.0000000000

-7.1115498543 12.3175656682 0.0000000000

0.0000000000 0.0000000000 20.0000000000

C N V O H

26 24 1 2 1

Direct

0.218070016 0.440780035 0.499069977

0.062070002 0.278739999 0.496780014

0.222890010 0.280580018 0.498079967

0.290069983 0.067319999 0.499079990

0.447570011 0.229569998 0.498330021

0.285530001 0.223930014 0.498220015

0.730030004 0.442330023 0.490399981

0.569119989 0.287820026 0.495970011

0.724250052 0.281050000 0.496950006

0.782749959 0.058449999 0.498829985

0.941950074 0.217230018 0.496780014

0.782970025 0.217080009 0.497469997

0.230999980 0.944719955 0.499069977

0.072490014 0.786059977 0.498149967

0.231380001 0.785690046 0.499340010

0.278950011 0.558160013 0.499740028

0.445360018 0.715070055 0.500999975

0.288500025 0.721960011 0.499800014

0.722620058 0.935780046 0.499949980

0.565429991 0.775270024 0.501480007

0.725560055 0.778010057 0.500570011

0.792510010 0.564140044 0.491030025

0.949839991 0.723680061 0.497319984

0.789749979 0.720539978 0.498129988

0.444610002 0.482439992 0.628819990

0.449320006 0.579909982 0.637969971

0.395709980 0.288390021 0.498210001

0.229910002 0.115490002 0.498499966

0.399329997 0.121559999 0.499060011

0.116329999 0.222859996 0.496290016

0.283340001 0.393070017 0.500370026

0.111260004 0.387820016 0.497620010

0.891160034 0.275780005 0.496210003

0.723060022 0.107979997 0.498869991

0.891250069 0.107830008 0.497949982

0.615790022 0.226120007 0.500769997

0.785799974 0.389849994 0.493219995

0.621079951 0.395750007 0.488999987

0.398150032 0.774750045 0.500299978

0.226940032 0.613250041 0.499609995

0.391860023 0.602270012 0.500649977

0.121990010 0.727280041 0.498570013

0.290420008 0.895290042 0.499669981

0.121880016 0.894900058 0.498340034

0.898199939 0.780690009 0.501999950

0.732179986 0.612550052 0.491759968

0.900969969 0.614920037 0.492469978

0.615659995 0.716050010 0.502120018

0.782270031 0.886110042 0.499659967

0.614350029 0.884019983 0.500489998

0.441489977 0.489069997 0.521600008

0.525850005 0.481440024 0.593020010

0.450280013 0.661130043 0.649730015

0.381859981 0.410590007 0.655060005

V-CHO-CHO

1.0

14.2230997086 0.0000000000 0.0000000000

-7.1115498543 12.3175656682 0.0000000000

0.0000000000 0.0000000000 20.0000000000

C N V O H

26 24 1 2 2

Direct

0.220840001 0.440409987 0.499390030

0.064470000 0.279060012 0.498029995

0.224770005 0.280580018 0.499590015

0.290489992 0.066179999 0.499559975

0.448250020 0.227720012 0.498290014

0.287030020 0.223230005 0.499160004

0.730210045 0.441650009 0.491079998

0.569800037 0.286370011 0.495870018

0.725589984 0.280889984 0.496600008

0.784949978 0.058960000 0.498189974

0.943789971 0.217639997 0.497599983

0.784990000 0.217570006 0.497209978

0.231089997 0.943429995 0.499730015

0.072619984 0.784619970 0.498360014

0.231450056 0.784570031 0.499630022

0.282190003 0.559189984 0.499700022

0.446999995 0.716449982 0.500139999

0.289640010 0.721710009 0.499740028

0.724500028 0.936389992 0.498580027

0.567070016 0.776480005 0.499429989

0.726719948 0.778309997 0.497650003

0.792430032 0.563359998 0.491210032

0.949949963 0.722490055 0.497070026

0.790210012 0.720130019 0.495629978

0.431070020 0.488830021 0.621590042

0.443109998 0.592090004 0.643169975

0.396900004 0.286630001 0.498589993

0.230619983 0.114729999 0.498959970

0.399589993 0.119570001 0.499520016

0.117650002 0.222829994 0.498509979

0.284740002 0.392420004 0.500710011

0.114050000 0.388460003 0.498000002

0.893389989 0.276369995 0.496899986

0.725459975 0.108609996 0.497849989

0.893519980 0.108470004 0.498149967

0.617039985 0.225120000 0.499539995

0.786560011 0.389659996 0.493540001

0.621269967 0.394530000 0.490150023

0.398840036 0.775530013 0.500029993

0.228710001 0.612620004 0.499249983

0.394110014 0.604219972 0.500289965

0.122230003 0.725889972 0.498479986

0.290430007 0.894029968 0.500259972

0.122010017 0.893470038 0.498909998

0.898650034 0.779970043 0.499609995

0.732300026 0.611970030 0.490250015

0.900799954 0.613669989 0.493790007

0.617010047 0.716960051 0.498699999

0.783840042 0.886520079 0.498199987

0.616129966 0.885089981 0.498820019

0.447009994 0.491690023 0.517579985

0.517870030 0.487050007 0.593760014

0.371250024 0.603079982 0.670170021

0.364930006 0.413390004 0.642829990

0.524490011 0.663699976 0.630690002

V-CHO-CH_2_O

1.0

14.2230997086 0.0000000000 0.0000000000

-7.1115498543 12.3175656682 0.0000000000

0.0000000000 0.0000000000 20.0000000000

C N V O H

26 24 1 2 3

Direct

0.223330027 0.449890005 0.500989962

0.069369999 0.285400006 0.502979994

0.234610007 0.293940020 0.506389999

0.287510006 0.070569999 0.498710012

0.448460017 0.226640006 0.490330029

0.293440001 0.232330007 0.501649952

0.728810003 0.441859983 0.493279982

0.570399965 0.284430000 0.489219999

0.728049992 0.283090005 0.498839998

0.788400004 0.062270004 0.501679993

0.948380019 0.221340009 0.503149986

0.789030010 0.221300020 0.500800037

0.223870008 0.947860037 0.497739983

0.065340040 0.789490012 0.498010015

0.223929975 0.789219958 0.491440010

0.278550016 0.567120013 0.493660021

0.442739968 0.728039957 0.493099976

0.283320018 0.728420031 0.489739990

0.723860027 0.939560037 0.501600027

0.561519975 0.783680043 0.499100018

0.717019986 0.776480005 0.507810020

0.786930023 0.563489992 0.496689987

0.942590047 0.725440061 0.499800014

0.780260043 0.717829986 0.506370020

0.477370002 0.447850001 0.654059982

0.425440035 0.513930046 0.669619989

0.401459983 0.288980011 0.491989994

0.232940015 0.123579999 0.506579971

0.396230031 0.118430002 0.492049980

0.127660007 0.234040011 0.504270029

0.292919995 0.407240028 0.510160017

0.116510010 0.394020008 0.497959995

0.897260046 0.279879989 0.502500010

0.729409967 0.112419998 0.499970007

0.897289977 0.111500008 0.503449965

0.619130050 0.225010000 0.495660019

0.786940005 0.392080036 0.500750017

0.621029984 0.392800001 0.484560013

0.393270008 0.785410003 0.490960026

0.223739991 0.619160003 0.491560030

0.390789962 0.616080002 0.492910004

0.114580021 0.730790055 0.492869997

0.282560006 0.898510026 0.492680025

0.114780009 0.898530001 0.501590014

0.888899968 0.779930015 0.506540012

0.723960011 0.609330029 0.502510023

0.895219998 0.616800006 0.494120026

0.606160009 0.718449997 0.507740021

0.777580027 0.884230034 0.506340027

0.616020038 0.891920011 0.495260000

0.452610010 0.528559994 0.529640007

0.502470031 0.445349982 0.594619989

0.432280010 0.576259985 0.614039993

0.492860004 0.401419994 0.692529964

0.463580030 0.565230018 0.714389992

0.339550001 0.456140013 0.684249973

V-CHO-CH_3_O

1.0

14.2230997086 0.0000000000 0.0000000000

-7.1115498543 12.3175656682 0.0000000000

0.0000000000 0.0000000000 20.0000000000

C N V O H

26 24 1 2 4

Direct

0.219750012 0.456830015 0.498640013

0.066140001 0.292440009 0.504670048

0.232340007 0.303160010 0.511640024

0.281109991 0.076200001 0.499669981

0.443390030 0.231020013 0.492869997

0.290400016 0.239530006 0.507179976

0.723249985 0.445950018 0.491610003

0.565309996 0.288030000 0.490779972

0.723200060 0.287829994 0.502309990

0.784289983 0.067150000 0.506299973

0.944119970 0.227000008 0.505959988

0.784579983 0.226500004 0.504920006

0.216570036 0.953440057 0.496089983

0.057979997 0.794879956 0.496039963

0.216430010 0.795030004 0.483909988

0.273679991 0.574249983 0.488679981

0.437050042 0.737620007 0.489980030

0.276579994 0.734889961 0.481080008

0.718749984 0.945020050 0.505490017

0.554400034 0.791360032 0.499989986

0.708130026 0.778949983 0.512290001

0.780170067 0.567730036 0.494630003

0.935100065 0.730240035 0.499779987

0.771700058 0.720630022 0.508470011

0.522490021 0.436040026 0.640619993

0.460180004 0.441159993 0.698869991

0.398080002 0.294660004 0.497799969

0.228210007 0.130590010 0.510309982

0.389529998 0.122819996 0.491829967

0.125180010 0.242239998 0.509850025

0.289199997 0.416190002 0.511940002

0.112420002 0.399930009 0.494789982

0.893219982 0.285570009 0.505089998

0.725579996 0.117889999 0.505299997

0.893830017 0.117380007 0.507279968

0.614100007 0.229420009 0.501090002

0.781649983 0.396919999 0.501739979

0.615790043 0.395669990 0.481489992

0.386550002 0.793429961 0.483869982

0.217950008 0.624790039 0.482579994

0.385520028 0.625600009 0.492110014

0.107040021 0.736610011 0.486679983

0.274850010 0.904389986 0.486740017

0.107690032 0.903730024 0.502379990

0.880650027 0.783210003 0.510020018

0.716340027 0.612659993 0.500959969

0.888350010 0.621800004 0.492159986

0.595510018 0.722140021 0.512239981

0.769190036 0.886170045 0.510550022

0.611230037 0.898870047 0.496470022

0.454320007 0.556020016 0.542630005

0.523810054 0.472799978 0.584070015

0.427980011 0.589079994 0.614949989

0.572390032 0.396810018 0.647499990

0.497030021 0.526359993 0.715590000

0.376879993 0.418020032 0.684010029

0.460419999 0.390669991 0.740220022

V-CH_2_O-CH_3_O

1.0

14.2230997086 0.0000000000 0.0000000000

-7.1115498543 12.3175656682 0.0000000000

0.0000000000 0.0000000000 20.0000000000

C N V O H

26 24 1 2 5

Direct

0.219899993 0.449390001 0.499769974

0.062010008 0.290700003 0.499810028

0.221410008 0.291599998 0.501399994

0.279599991 0.070320002 0.495809984

0.438730003 0.228010003 0.491849995

0.281630000 0.229700012 0.499079990

0.718280045 0.447720006 0.495609999

0.561029952 0.288930014 0.492059994

0.719800021 0.290140015 0.500760031

0.780969984 0.070299997 0.499940014

0.939530039 0.228960014 0.500269985

0.781490002 0.229360005 0.501709986

0.217450022 0.947830074 0.494670010

0.058950018 0.789800018 0.494019985

0.217149988 0.788990008 0.490899992

0.279720020 0.570610013 0.499350023

0.439979980 0.736439988 0.504899979

0.279350019 0.730279986 0.492140007

0.718490063 0.949690029 0.499949980

0.557020028 0.794940037 0.507019997

0.717400040 0.788909952 0.503799963

0.778020020 0.569860007 0.496589994

0.936469971 0.728839978 0.495639992

0.777760025 0.728089973 0.500890017

0.516529973 0.429610007 0.638880014

0.456849978 0.434869995 0.700559998

0.389870003 0.287889998 0.493750000

0.222000012 0.121119999 0.501539993

0.388190004 0.119439997 0.491330004

0.113080003 0.232779994 0.500559998

0.280310006 0.401039997 0.502520037

0.111380018 0.399230019 0.498230028

0.890410045 0.288599995 0.501760006

0.722580022 0.121159998 0.501580000

0.890159980 0.120399995 0.498820019

0.611060026 0.230749997 0.497580004

0.777600022 0.398930040 0.502640009

0.610339965 0.397500020 0.488189983

0.386559997 0.790290034 0.497480011

0.223350014 0.619540000 0.489190006

0.389470008 0.623510028 0.511730003

0.108010014 0.730320014 0.490750027

0.275960036 0.898200020 0.492220020

0.108540006 0.898490050 0.496460009

0.886470044 0.787240073 0.499399996

0.718360019 0.619320000 0.500519991

0.886200038 0.619910009 0.493440008

0.606879996 0.731679965 0.514699984

0.774129983 0.894720047 0.496560001

0.610959996 0.902450052 0.502029991

0.496930004 0.600619992 0.564350033

0.503960015 0.484130002 0.582940006

0.494170027 0.660380038 0.632649994

0.604340058 0.466439989 0.648869991

0.485419974 0.519519976 0.714809990

0.368970004 0.394430007 0.691169977

0.471149992 0.393429999 0.742479992

0.485739991 0.344799992 0.623059988

V-CH_2_OH-CH_3_O

1.0

14.2230997086 0.0000000000 0.0000000000

-7.1115498543 12.3175656682 0.0000000000

0.0000000000 0.0000000000 20.0000000000

C N V O H

26 24 1 2 6

Direct

0.205730004 0.462250000 0.513050032

0.052870011 0.297080025 0.517030001

0.219110011 0.308990012 0.526660013

0.267650008 0.081279998 0.522560024

0.430599971 0.234899997 0.523150015

0.277440000 0.245369996 0.526040030

0.712460021 0.451000032 0.521089983

0.552049988 0.291209994 0.523299980

0.710749976 0.291450008 0.516129971

0.771329949 0.071220007 0.517499971

0.931099978 0.231120006 0.517099953

0.771610023 0.230290002 0.516300011

0.203130012 0.958980049 0.517400026

0.044590032 0.800710056 0.516960049

0.202549983 0.801029971 0.501779985

0.259070014 0.579999987 0.503520012

0.422729986 0.743179974 0.503090000

0.262349973 0.740729971 0.496780014

0.705640053 0.949280070 0.516839981

0.540760008 0.796460024 0.512350035

0.693769975 0.783000029 0.525759983

0.769009998 0.572449993 0.520110035

0.922249979 0.735710036 0.521879959

0.757670013 0.724920005 0.524529982

0.484809987 0.430300009 0.658770037

0.526310045 0.502160022 0.720200014

0.386190001 0.299650015 0.522870016

0.214420002 0.136509999 0.526550007

0.376740011 0.126900005 0.522650003

0.112070009 0.247310007 0.523050022

0.275209985 0.421759995 0.527059984

0.098640013 0.404830015 0.508099985

0.880190007 0.289570000 0.516079998

0.712459988 0.121700001 0.516930008

0.880909939 0.121449999 0.518190002

0.601490049 0.233010002 0.517109966

0.769699926 0.400759994 0.514190006

0.603960025 0.400070030 0.529290009

0.372610033 0.799490009 0.497729969

0.203240010 0.630720015 0.498180008

0.370630017 0.631790014 0.506299973

0.093300030 0.742680047 0.505249977

0.261249985 0.910260036 0.505919981

0.094459994 0.909280004 0.524670029

0.866490025 0.788240003 0.526999998

0.704080021 0.616610007 0.518549967

0.876999950 0.626640045 0.521000004

0.581150039 0.727150046 0.526139975

0.755430057 0.890240066 0.522580051

0.597950000 0.903590042 0.507550001

0.437429993 0.563290006 0.560039997

0.521780008 0.495540005 0.598299980

0.404870004 0.590050000 0.632040024

0.395440002 0.382520000 0.658290005

0.615499961 0.550199982 0.720079994

0.493559986 0.557500013 0.722100019

0.516010010 0.373360014 0.656939983

0.551519990 0.460480011 0.564820004

0.499810023 0.451290023 0.765429974

V-O

1.0

14.2230997086 0.0000000000 0.0000000000

-7.1115498543 12.3175656682 0.0000000000

0.0000000000 0.0000000000 20.0000000000

C N V O

24 24 1 1

Direct

0.212270007 0.426930017 0.503109980

0.054720002 0.265520019 0.502489996

0.213970022 0.264640018 0.501460028

0.284619997 0.054770001 0.500150013

0.441440007 0.218940003 0.501030016

0.277020018 0.210020011 0.500790024

0.724319968 0.430990011 0.499000025

0.562650031 0.276710002 0.500810051

0.717299979 0.268710001 0.502379990

0.775919947 0.046190002 0.503870010

0.935050012 0.205030001 0.502900028

0.775829974 0.204559999 0.503099966

0.227269984 0.932600013 0.500400019

0.068500014 0.773410069 0.500829983

0.227339993 0.773219993 0.501779985

0.274670014 0.544380025 0.503269958

0.440380012 0.699620033 0.503030014

0.284209969 0.708190010 0.502340031

0.716929948 0.923439989 0.503940010

0.561390038 0.761520076 0.502540016

0.722440011 0.767290017 0.499860001

0.787760046 0.552489988 0.498390007

0.945850022 0.711450022 0.500339985

0.786599990 0.709969986 0.498820019

0.388489977 0.277740011 0.501550007

0.223690012 0.101960006 0.500040007

0.394320008 0.111560002 0.500329971

0.107610002 0.208470004 0.501299953

0.275850008 0.377559991 0.502260017

0.105880010 0.375330027 0.503090000

0.883989999 0.263449993 0.502640009

0.715789933 0.095310007 0.503819990

0.884190010 0.095520000 0.503400040

0.608720021 0.214279992 0.503310013

0.779259944 0.377779991 0.501009989

0.615069945 0.385220005 0.497720003

0.394000014 0.760420037 0.502139997

0.222470013 0.599960030 0.502890015

0.386539996 0.587710017 0.503600025

0.118120013 0.714530023 0.501719999

0.286699976 0.882689995 0.501169968

0.117969975 0.882059996 0.500040007

0.895070029 0.769660041 0.500440025

0.728129965 0.601469991 0.496950006

0.896249997 0.602439996 0.500000000

0.612949995 0.704389966 0.498540020

0.777950009 0.875410055 0.502200031

0.608439936 0.869989993 0.505450010

0.433279975 0.459100007 0.518369961

0.455260007 0.469639998 0.597819996

V-OH

1.0

14.2230997086 0.0000000000 0.0000000000

-7.1115498543 12.3175656682 0.0000000000

0.0000000000 0.0000000000 20.0000000000

C N V O H

24 24 1 1 1

Direct

0.214009996 0.429080002 0.506579971

0.059090002 0.266100001 0.503299999

0.221640010 0.270460014 0.503310013

0.282970018 0.053300001 0.500259972

0.442110000 0.213129995 0.501230001

0.282559993 0.212190011 0.501169968

0.723660025 0.427210020 0.500559998

0.563730004 0.271230014 0.501490021

0.720029992 0.266699998 0.503220034

0.779440025 0.045040001 0.502680016

0.938839978 0.203810012 0.503260040

0.779720025 0.203669991 0.503590012

0.222450003 0.930710018 0.500029993

0.064259999 0.771910058 0.500390005

0.223270000 0.772029987 0.502789974

0.272740000 0.546440005 0.505739975

0.439189983 0.704929998 0.503409958

0.281080002 0.709580002 0.503719997

0.717630064 0.922400030 0.502290010

0.558640016 0.763390019 0.502960014

0.717360025 0.762969994 0.501470041

0.784660002 0.548890008 0.499940014

0.941700009 0.709130014 0.500220013

0.781020012 0.705050005 0.500289965

0.392560008 0.273730013 0.500850010

0.224820002 0.103349998 0.500479984

0.392430023 0.105170002 0.501200008

0.114930003 0.212270009 0.501339960

0.281540025 0.383450016 0.508099985

0.107479995 0.375250009 0.504600000

0.887930015 0.262369987 0.503960037

0.719919958 0.094700003 0.502990007

0.888019981 0.094260003 0.502619982

0.611199973 0.210209990 0.503419971

0.780530010 0.375669996 0.502200031

0.614810021 0.379710015 0.499609995

0.390860026 0.763360056 0.502900028

0.220100026 0.600470022 0.505360031

0.385700003 0.591820027 0.504580021

0.113779986 0.713359993 0.502699947

0.281920007 0.881610008 0.501850033

0.113319992 0.880900031 0.498589993

0.889559982 0.765860075 0.500460052

0.723479970 0.596520008 0.499529982

0.893079984 0.600149990 0.500309992

0.607360019 0.702220006 0.502619982

0.775569978 0.870960037 0.501359987

0.609449987 0.872189994 0.502729988

0.440510015 0.487950021 0.519589996

0.467479984 0.494859990 0.608059978

0.417020001 0.447580024 0.642119980

V-H_2_O

1.0

14.2230997086 0.0000000000 0.0000000000

-7.1115498543 12.3175656682 0.0000000000

0.0000000000 0.0000000000 20.0000000000

C N V O H

24 24 1 1 2

Direct

0.210280020 0.421330022 0.499399996

0.048790005 0.263260014 0.497970009

0.203739996 0.255019998 0.499709988

0.275460004 0.044390003 0.498499966

0.431999998 0.208750007 0.499779987

0.264570005 0.197929994 0.500240040

0.711799996 0.427480036 0.512220001

0.551960007 0.270289991 0.501500034

0.709640040 0.267230003 0.507509995

0.770060032 0.046720003 0.504209995

0.928990014 0.205489995 0.499480009

0.769900028 0.205080016 0.505310011

0.218260014 0.923829973 0.499930000

0.059209987 0.764869977 0.504329967

0.217509996 0.764069956 0.501820040

0.273560000 0.540139981 0.501100016

0.435230016 0.697550027 0.506099987

0.277129996 0.701259949 0.502399969

0.712090038 0.924460049 0.506789970

0.556770014 0.761979974 0.508990002

0.719109982 0.769949994 0.516919994

0.777039970 0.548800002 0.516669989

0.937100009 0.705829974 0.507560015

0.781469991 0.710839999 0.519490004

0.377430011 0.266779996 0.502510023

0.213199993 0.091339998 0.498650026

0.385129993 0.101790003 0.497630024

0.097339999 0.202120003 0.498409986

0.269499996 0.367950017 0.500850010

0.103600008 0.373020007 0.497870016

0.878200054 0.264250014 0.501699972

0.710060027 0.095770002 0.507179976

0.878010001 0.096070000 0.500010014

0.601689986 0.211350000 0.501770020

0.768270018 0.375990027 0.515339947

0.602339978 0.380470008 0.503950024

0.386599989 0.756710037 0.505680037

0.218049991 0.593029970 0.500010014

0.384530022 0.586359976 0.504100037

0.108210025 0.704860007 0.504250002

0.276959994 0.872850033 0.499270010

0.108860013 0.873160038 0.502589989

0.888189970 0.766129974 0.509570026

0.722339996 0.601860014 0.528620005

0.883789999 0.596589999 0.506250000

0.609810006 0.706860022 0.517019987

0.774259996 0.877910073 0.512889957

0.603330008 0.870010046 0.502960014

0.428460010 0.428830000 0.511049986

0.532000014 0.505620020 0.590569973

0.607779986 0.544420014 0.566730022

0.523320052 0.566030039 0.605799961

Zn/g−CN

1.0

14.2230997086 0.0000000000 0.0000000000

-7.1115498543 12.3175656682 0.0000000000

0.0000000000 0.0000000000 20.0000000000

C N Zn

24 24 1

Direct

0.226909992 0.440309994 0.500000000

0.063460009 0.282880012 0.500000000

0.219369994 0.276000005 0.500000000

0.276699996 0.054570001 0.500000000

0.437050027 0.212419998 0.500000000

0.274320015 0.212480002 0.500000000

0.712059992 0.440510019 0.500000000

0.555419983 0.276000005 0.500000000

0.718450024 0.283160015 0.500000000

0.782500024 0.066370002 0.500000000

0.941750009 0.224990005 0.500000000

0.782159984 0.225169997 0.500000000

0.216869986 0.935050016 0.500000000

0.058000006 0.776100008 0.500000000

0.216550002 0.776080033 0.500000000

0.285569990 0.561180011 0.500000000

0.441400030 0.722180049 0.500000000

0.279940028 0.717040030 0.500000000

0.721150009 0.943939986 0.500000000

0.562650028 0.785099998 0.500000000

0.720730007 0.785150014 0.500000000

0.773940020 0.561330020 0.500000000

0.935569992 0.717140061 0.500000000

0.779080037 0.722200025 0.500000000

0.385659992 0.272489992 0.500000000

0.217920017 0.105860004 0.500000000

0.386689993 0.105769999 0.500000000

0.111589998 0.222350004 0.500000000

0.285830017 0.387700009 0.500000000

0.119570012 0.392700008 0.500000000

0.891640036 0.284320000 0.500000000

0.723320004 0.115810005 0.500000000

0.891120059 0.115620007 0.500000000

0.609759972 0.222609994 0.500000000

0.771960074 0.393070017 0.500000000

0.600489956 0.387800002 0.500000000

0.389099969 0.777999992 0.500000000

0.224280001 0.608470043 0.500000000

0.394510004 0.612730023 0.500000000

0.107270021 0.716120001 0.500000000

0.275509984 0.884289960 0.500000000

0.107400028 0.884380004 0.500000000

0.887249998 0.778060073 0.500000000

0.716489982 0.612760025 0.500000000

0.882550081 0.608600038 0.500000000

0.612060038 0.725830046 0.500000000

0.779839993 0.893580057 0.500000000

0.612150045 0.893600032 0.500000000

0.466259994 0.432000007 0.500000000

Zn-CO

1.0

14.2230997086 0.0000000000 0.0000000000

-7.1115498543 12.3175656682 0.0000000000

0.0000000000 0.0000000000 20.0000000000

C N Zn O

25 24 1 1

Direct

0.216560019 0.433739993 0.504469967

0.055929995 0.274610014 0.498390007

0.211830017 0.268740002 0.500390005

0.276410011 0.053590002 0.494589996

0.435730011 0.214100001 0.501309967

0.270729994 0.209540002 0.499489975

0.715139957 0.435160025 0.506349993

0.556399987 0.275690000 0.502579975

0.715729979 0.276710002 0.500930023

0.776839952 0.057470001 0.499359989

0.936000013 0.216140005 0.498360014

0.777030007 0.215950007 0.499650002

0.217300033 0.933560070 0.494299984

0.058240029 0.774840011 0.496960020

0.216169998 0.774090006 0.497870016

0.277379997 0.554440026 0.504719973

0.436310012 0.713810058 0.504859972

0.277019996 0.712919993 0.500209999

0.716469985 0.935430013 0.500639963

0.558080004 0.775989989 0.505310011

0.718339967 0.777819981 0.507130051

0.776979970 0.556800023 0.507200003

0.936270011 0.714869992 0.499599981

0.779060039 0.717149972 0.507499981

0.545310009 0.547630011 0.573649979

0.382809999 0.272700023 0.505539989

0.216690009 0.102810005 0.494299984

0.387100013 0.107770004 0.496059990

0.104980005 0.214800010 0.496140003

0.275290011 0.380780014 0.507210016

0.110140009 0.385160001 0.500649977

0.885499997 0.275170001 0.498959970

0.717169988 0.106839998 0.499779987

0.885060007 0.106610000 0.498629999

0.607129963 0.218109999 0.499160004

0.773800039 0.385970010 0.503859997

0.605369993 0.385060008 0.507019997

0.386450011 0.771730047 0.501179981

0.218930003 0.604370020 0.501339960

0.386800003 0.604219972 0.507579994

0.107130010 0.714710034 0.498899984

0.275760019 0.882520049 0.495770025

0.107730008 0.883000000 0.494370031

0.886770012 0.774330020 0.500930023

0.719550059 0.608080058 0.512919998

0.884820037 0.605800001 0.500659990

0.609130015 0.718269986 0.510319996

0.775809974 0.885839987 0.502530003

0.607310000 0.884060011 0.500230026

0.433109992 0.430059995 0.517689991

0.578310002 0.584770036 0.625509977

Zn-CHO

1.0

14.2230997086 0.0000000000 0.0000000000

-7.1115498543 12.3175656682 0.0000000000

0.0000000000 0.0000000000 20.0000000000

C N Zn O H

25 24 1 1 1

Direct

0.210930002 0.428579998 0.499740028

0.051040011 0.269460006 0.495300007

0.207930000 0.265690003 0.497669983

0.272299999 0.050240000 0.496430016

0.430720000 0.210620008 0.502510023

0.267870008 0.206719991 0.498099995

0.710940024 0.430719996 0.504349995

0.552410002 0.271930023 0.504230022

0.710819967 0.271780014 0.504410028

0.771709942 0.052049997 0.506519985

0.930329992 0.210180008 0.497779989

0.772109994 0.210650010 0.503979969

0.212590003 0.929159991 0.497130013

0.053729986 0.770940013 0.502680016

0.211729991 0.770139992 0.499959993

0.272070005 0.549960045 0.500570011

0.431289999 0.708500016 0.502449989

0.272250004 0.708380009 0.500169992

0.710770044 0.929610056 0.507999992

0.553240015 0.770320003 0.503629971

0.711860014 0.771610040 0.503789997

0.772510023 0.552630009 0.503979969

0.931429979 0.710440027 0.504790020

0.773109986 0.711040062 0.503279972

0.510009989 0.498010022 0.619689989

0.378719974 0.269020006 0.504080009

0.212750010 0.099080001 0.494110012

0.382500015 0.103430006 0.499049997

0.100440007 0.210250019 0.493849993

0.269689999 0.376920005 0.503269958

0.104049993 0.379889987 0.495760012

0.880230009 0.269370001 0.497940016

0.712819956 0.102130010 0.509410000

0.879719979 0.101080006 0.501009989

0.602090028 0.213090006 0.504710007

0.769280025 0.380699996 0.503940010

0.601600017 0.380739985 0.504469967

0.381280055 0.766740075 0.500999975

0.213389999 0.599760006 0.499690008

0.380970010 0.599030014 0.502489996

0.103050035 0.711270011 0.503639984

0.271259995 0.878650014 0.496499968

0.103520047 0.879200034 0.499620008

0.881680037 0.769490018 0.504710007

0.713630019 0.602219996 0.501079988

0.881020049 0.601670054 0.506139994

0.603160028 0.711920063 0.499620008

0.769940033 0.879930064 0.507849979

0.601959996 0.878770021 0.507900000

0.429830043 0.433660014 0.533659983

0.582610006 0.590770004 0.629570007

0.484169994 0.439900035 0.663420010

Zn-CH_2_O

1.0

14.2230997086 0.0000000000 0.0000000000

-7.1115498543 12.3175656682 0.0000000000

0.0000000000 0.0000000000 20.0000000000

C N Zn O H

25 24 1 1 2

Direct

0.210779996 0.428519995 0.499429989

0.050340011 0.269370001 0.501640034

0.206980003 0.264800015 0.496980000

0.270969997 0.049110002 0.501060009

0.430159982 0.209680004 0.501070023

0.266389991 0.205789994 0.496990013

0.709030002 0.430270008 0.506699991

0.550580012 0.270819996 0.503989983

0.709399942 0.271250009 0.507810020

0.770660001 0.051930000 0.507669973

0.929720016 0.210370006 0.503600025

0.770950006 0.210439997 0.507420015

0.211750007 0.928589996 0.501290035

0.052920017 0.769750008 0.502569962

0.211110005 0.769360023 0.502369976

0.271960005 0.549210039 0.500729990

0.431129999 0.707690006 0.503180027

0.272000022 0.707820001 0.502729988

0.710070034 0.929520012 0.507749987

0.552820034 0.769889990 0.504410028

0.711769951 0.771769998 0.504849958

0.770960013 0.551980035 0.506529999

0.930439938 0.709330000 0.504139996

0.772620052 0.710910068 0.504790020

0.456910023 0.454809986 0.638920021

0.378529982 0.269190010 0.495779991

0.211539996 0.098520003 0.499709988

0.381240023 0.102860001 0.503230000

0.099639990 0.209919999 0.500289965

0.270159984 0.376820012 0.495330000

0.104019996 0.379759992 0.502150011

0.879440031 0.269480001 0.504329967

0.711370045 0.101500002 0.509720039

0.878880086 0.101060001 0.504640007

0.600909995 0.212720016 0.506470013

0.767309955 0.380549987 0.508650017

0.599399999 0.380299985 0.503380013

0.381170010 0.765850009 0.503779984

0.213549993 0.599309979 0.502040005

0.381229994 0.598090010 0.500699997

0.102120004 0.709980051 0.502919960

0.270450018 0.877880033 0.501810026

0.102350013 0.878020015 0.501470041

0.881039977 0.768800016 0.503380013

0.712360018 0.601979982 0.505720043

0.879490043 0.600580003 0.506250000

0.602999965 0.711560042 0.501909971

0.769599965 0.880129972 0.507579994

0.601279954 0.878359984 0.507430029

0.431380038 0.430400041 0.518019962

0.513560010 0.554480015 0.646850014

0.366490026 0.412190011 0.643959999

0.493589999 0.401280011 0.637750053

Zn-CH_3_O

1.0

14.2230997086 0.0000000000 0.0000000000

-7.1115498543 12.3175656682 0.0000000000

0.0000000000 0.0000000000 20.0000000000

C N Zn O H

25 24 1 1 3

Direct

0.204879997 0.431780007 0.499590015

0.048760004 0.270680014 0.500339985

0.209450001 0.273160018 0.505480003

0.269829990 0.053780001 0.503669977

0.429010003 0.212350007 0.503799963

0.270639990 0.212639998 0.505729961

0.709600017 0.429560030 0.503240013

0.550859991 0.272170018 0.504019976

0.708310027 0.270460014 0.507439995

0.768959957 0.050220000 0.504499960

0.927580028 0.208690004 0.501990032

0.768989989 0.208690004 0.506529999

0.208599993 0.931249972 0.502180004

0.050150010 0.772810033 0.503170013

0.208730026 0.772840074 0.497379971

0.263800013 0.550660035 0.497959995

0.426920015 0.710489965 0.500790024

0.267980034 0.711380031 0.495739985

0.707189995 0.927830003 0.504059982

0.548420005 0.769909965 0.502709961

0.706319987 0.768410032 0.504119968

0.770220019 0.551419989 0.502709961

0.927870009 0.710870039 0.504829979

0.768329971 0.708510003 0.503709984

0.438820012 0.404330011 0.652629948

0.379350023 0.271990007 0.504769993

0.211490017 0.104110001 0.505909967

0.378699995 0.104009998 0.502510023

0.102219997 0.214009995 0.503079987

0.267309991 0.383430002 0.506229973

0.097100006 0.378860016 0.496350002

0.877269975 0.267459991 0.504069996

0.709719986 0.099710009 0.507200003

0.877529990 0.099510003 0.501860046

0.599549975 0.212400004 0.507870007

0.767730028 0.379300016 0.506640005

0.600889992 0.380549987 0.500119972

0.378410026 0.768879995 0.497280025

0.208529989 0.602900049 0.494169998

0.374960007 0.600290011 0.502890015

0.099600009 0.714140039 0.499309969

0.267799991 0.882059996 0.498230028

0.099850008 0.881820059 0.505079985

0.876870015 0.768610018 0.506010008

0.710090010 0.600090025 0.500460052

0.878690082 0.602040024 0.504679966

0.596880004 0.709519998 0.503669977

0.764980025 0.876739966 0.504369974

0.598550076 0.878320033 0.502900028

0.430880014 0.498530001 0.532259989

0.491809980 0.499250005 0.613009977

0.348800007 0.364039993 0.650860023

0.461030019 0.342770014 0.637839985

0.463109995 0.426310006 0.705399990

Zn-CH_3_O

1.0

14.2230997086 0.0000000000 0.0000000000

-7.1115498543 12.3175656682 0.0000000000

0.0000000000 0.0000000000 20.0000000000

C N Zn O H

25 24 1 1 4

Direct

0.193590008 0.428810025 0.502139997

0.038020003 0.266620019 0.499659967

0.198500002 0.268319997 0.504800034

0.263590014 0.053100001 0.501460028

0.422100010 0.213269997 0.504220009

0.261550009 0.210780005 0.504600000

0.705029993 0.428320009 0.514370012

0.544099960 0.272530000 0.505480003

0.700459970 0.267710012 0.508400011

0.759559976 0.046459999 0.506969976

0.918360009 0.205140001 0.501580000

0.759509983 0.204799993 0.506519985

0.203050028 0.931000048 0.501250029

0.044530012 0.772409984 0.504300022

0.203339981 0.772099979 0.499529982

0.253740015 0.547249975 0.501429987

0.419559993 0.706189995 0.504040003

0.261510009 0.709969986 0.498640013

0.699099995 0.924470036 0.509410000

0.540769987 0.765090016 0.506589985

0.699840043 0.766129974 0.516459990

0.766789939 0.549539981 0.518670034

0.922849997 0.710270004 0.508029985

0.763359998 0.708109954 0.519869995

0.503110012 0.430210004 0.640269995

0.371269989 0.272379991 0.507110023

0.204410007 0.102499999 0.502219963

0.373039995 0.105329999 0.501230001

0.092190000 0.210079996 0.500450039

0.255899999 0.379370008 0.509299994

0.086809998 0.375739987 0.497559977

0.867500011 0.263680020 0.502929974

0.699689954 0.095730003 0.508790016

0.867879985 0.095630000 0.503459978

0.591669991 0.211730016 0.503889990

0.761270004 0.376200001 0.515150023

0.596059981 0.381410012 0.509220028

0.372209999 0.765419997 0.499709988

0.200580000 0.601170012 0.497539997

0.365630006 0.595190020 0.507170010

0.093990020 0.713500053 0.502170038

0.262370004 0.881690065 0.498699999

0.094020030 0.881590033 0.504199982

0.869979968 0.766670006 0.509159994

0.708090010 0.599540006 0.530240011

0.873269977 0.600960038 0.508069992

0.590399982 0.705410027 0.511229992

0.757480053 0.874290040 0.516779995

0.590569972 0.873390065 0.504519987

0.411360004 0.482990012 0.523069954

0.519659989 0.509710016 0.589909983

0.419439998 0.394650007 0.658540010

0.515779978 0.365929988 0.619570017

0.559680001 0.471370035 0.681799984

0.595720053 0.541650019 0.565520000

Zn-CHO-CO

1.0

14.2230997086 0.0000000000 0.0000000000

-7.1115498543 12.3175656682 0.0000000000

0.0000000000 0.0000000000 20.0000000000

C N Zn O H

26 24 1 2 1

Direct

0.227499998 0.439170004 0.499909973

0.066409996 0.281050000 0.496679974

0.222210009 0.275599995 0.493160009

0.285419997 0.058870000 0.492729998

0.444260001 0.219060010 0.492170000

0.280900016 0.215490012 0.491359997

0.723539997 0.440430001 0.492370033

0.565520023 0.280990016 0.492509985

0.724420026 0.281839995 0.494480038

0.786940021 0.063249997 0.500230026

0.945460023 0.222080008 0.497640038

0.786450068 0.221420007 0.496360016

0.226519997 0.938149993 0.495009995

0.068250012 0.779170022 0.499240017

0.227170008 0.779980031 0.499559975

0.289000020 0.560720035 0.501299953

0.447590004 0.719240031 0.499849987

0.288629986 0.719039967 0.500450039

0.726629995 0.940820034 0.501309967

0.569220027 0.781450002 0.499709988

0.728179985 0.783110048 0.497230005

0.785870008 0.562290038 0.493079996

0.945819946 0.718829993 0.498880005

0.788640046 0.721690033 0.495860004

0.446970004 0.506529983 0.645569992

0.446890036 0.600040048 0.650320005

0.392370003 0.277520011 0.492049980

0.225660007 0.107789999 0.491310024

0.395799999 0.112000002 0.492539978

0.114449999 0.220850012 0.492370033

0.285279992 0.386730004 0.497149992

0.120239989 0.391199997 0.500109959

0.895110026 0.280959995 0.495709991

0.727339984 0.112599999 0.498409986

0.895259949 0.113030002 0.500190020

0.615760068 0.222809999 0.494409990

0.782100024 0.390740021 0.493190002

0.614020022 0.390000003 0.491529989

0.397300027 0.777340030 0.498659992

0.230120022 0.610230044 0.502479982

0.397820014 0.609820006 0.500249958

0.118249977 0.720229973 0.501730013

0.285930035 0.888430050 0.496169996

0.117340028 0.887380026 0.495940018

0.896930017 0.778760062 0.500339985

0.728169993 0.613080018 0.490649986

0.894320079 0.610109998 0.496630001

0.619370053 0.723160082 0.496029997

0.786270048 0.891600018 0.499510002

0.617889981 0.889949959 0.502690029

0.449010019 0.437389989 0.517000008

0.511550027 0.493140018 0.600629997

0.448920019 0.684170011 0.653940010

0.395960025 0.441800018 0.680870008

Zn-CHO-CHO

1.0

14.2230997086 0.0000000000 0.0000000000

-7.1115498543 12.3175656682 0.0000000000

0.0000000000 0.0000000000 20.0000000000

C N Zn O H

26 24 1 2 2

Direct

0.226859994 0.439429994 0.497919989

0.065559997 0.281050000 0.496029997

0.221369995 0.275460012 0.489860010

0.285489987 0.058810001 0.493690014

0.444310018 0.219359989 0.495830011

0.280719988 0.215290007 0.489890003

0.722840040 0.440610012 0.498859978

0.564660026 0.280990016 0.498040009

0.723810025 0.281899999 0.497640038

0.786379992 0.063350000 0.500010014

0.945000040 0.222270006 0.496910000

0.785809998 0.221560009 0.497870016

0.226549997 0.938260012 0.495770025

0.068009994 0.779259989 0.498969984

0.226569995 0.779550019 0.500469971

0.288250026 0.559840035 0.500320005

0.447060006 0.718640073 0.499209976

0.287779997 0.718300027 0.501480007

0.726130021 0.940869973 0.501189995

0.568770006 0.781060018 0.499779987

0.728010004 0.783190027 0.500590038

0.785340050 0.562330027 0.499199963

0.945630000 0.719169962 0.499340010

0.788410042 0.722000039 0.500150013

0.394949985 0.474350005 0.654039955

0.397330002 0.577539997 0.661590004

0.391969992 0.278039990 0.492469978

0.225580016 0.108180003 0.489949989

0.395320009 0.112340010 0.496670008

0.114359997 0.220620005 0.491470003

0.284880032 0.386730004 0.490520000

0.119709998 0.390470005 0.500969982

0.894350032 0.281019999 0.496269989

0.726569988 0.112499996 0.500050020

0.894639995 0.112960001 0.498460007

0.615320003 0.223079996 0.497550011

0.781430005 0.391119979 0.497459984

0.613139950 0.390430016 0.499580002

0.397060047 0.776850014 0.500180006

0.229690020 0.609980003 0.502860022

0.397570020 0.609199996 0.497709990

0.117490008 0.719899992 0.501440001

0.285630008 0.888000037 0.497919989

0.117270017 0.887520009 0.495849991

0.896839964 0.779259989 0.500010014

0.727410057 0.613000001 0.499810028

0.893890050 0.610329998 0.498849964

0.619070052 0.722780007 0.499489975

0.785819996 0.891670009 0.501649952

0.617250004 0.889630043 0.500509977

0.443839978 0.437130000 0.514209986

0.451230007 0.457230026 0.611079979

0.343780030 0.591799974 0.705110025

0.342300004 0.408499986 0.688819981

0.452540012 0.643100024 0.626590014

Zn-CHO-CH_2_O

1.0

14.2230997086 0.0000000000 0.0000000000

-7.1115498543 12.3175656682 0.0000000000

0.0000000000 0.0000000000 20.0000000000

C N Zn O H

26 24 1 2 3

Direct

0.217110015 0.439410018 0.504290009

0.060830004 0.278450027 0.500240040

0.220730011 0.279620000 0.508319950

0.282310025 0.061600001 0.506770039

0.441289991 0.220469996 0.506869984

0.282249968 0.219889994 0.508769989

0.721870035 0.438389997 0.502579975

0.563240006 0.280629995 0.505410004

0.720920026 0.279240022 0.500890017

0.781059963 0.058860002 0.503020000

0.940130040 0.217230018 0.499620008

0.781490024 0.217470013 0.500430012

0.221339991 0.939339999 0.505420017

0.062840022 0.780860032 0.505010033

0.221500012 0.780860032 0.501569986

0.276840009 0.558780024 0.504129982

0.439610010 0.717290032 0.505130005

0.281040039 0.719100048 0.500240040

0.719630047 0.936380082 0.504180002

0.561479991 0.777710039 0.503220034

0.719510017 0.777730014 0.498050022

0.782780020 0.560280035 0.501219988

0.940489999 0.719230043 0.504890013

0.781370025 0.717600037 0.498330021

0.447840019 0.404430004 0.679120016

0.547219996 0.511590024 0.664289999

0.391270019 0.279769989 0.508270025

0.223639984 0.111480004 0.508409977

0.391339986 0.112199998 0.505850029

0.114050008 0.221470004 0.502950001

0.278640003 0.390210016 0.511709976

0.110129998 0.387340027 0.498439980

0.889700019 0.276100018 0.498340034

0.721780052 0.108480001 0.502929974

0.889579985 0.107960003 0.501679993

0.612060047 0.220990014 0.502810001

0.780019985 0.388060031 0.499700022

0.613079996 0.389270012 0.506500006

0.391120010 0.776030017 0.500279999

0.221120010 0.610660017 0.499069977

0.387100039 0.607430007 0.510590029

0.112400007 0.722039990 0.502540016

0.280549989 0.889970012 0.502640009

0.112500028 0.889770026 0.506929970

0.889739988 0.777230011 0.502839994

0.723259979 0.609049987 0.494740009

0.891040069 0.610490033 0.506649971

0.610430050 0.718640073 0.496099997

0.778000054 0.886050038 0.500989962

0.611060036 0.886089989 0.508090019

0.432829994 0.489340013 0.544700050

0.368800033 0.363110015 0.641520023

0.544859997 0.562590017 0.606509972

0.448350012 0.362360009 0.725990009

0.616370034 0.495050027 0.666109991

0.559459989 0.562749975 0.709510040

Zn-CHO-CH_2_OH

1.0

14.2230997086 0.0000000000 0.0000000000

-7.1115498543 12.3175656682 0.0000000000

0.0000000000 0.0000000000 20.0000000000

C N Zn O H

26 24 1 2 4

Direct

0.225229988 0.444610003 0.502650023

0.067250005 0.284710004 0.502729988

0.227720020 0.286709999 0.504890013

0.283510000 0.064230005 0.496799994

0.443839993 0.221560009 0.496899986

0.287129998 0.224480014 0.502870035

0.722800003 0.440730019 0.498950005

0.565419984 0.281630022 0.496740007

0.724699954 0.282940015 0.501319981

0.786490050 0.063409999 0.504010010

0.945240082 0.222690011 0.503240013

0.786510016 0.222420015 0.502579975

0.221150028 0.941910086 0.495870018

0.062700023 0.783400001 0.497660017

0.221700026 0.783230055 0.495459986

0.283509995 0.564869997 0.499450016

0.444290019 0.729260081 0.498210001

0.282789995 0.724439976 0.495900011

0.724910037 0.943400032 0.504300022

0.562659959 0.787610004 0.502769995

0.721889992 0.781959993 0.508710003

0.781869999 0.562430020 0.500349998

0.940579994 0.722119968 0.499740028

0.781449979 0.720779993 0.507049990

0.471399993 0.474350005 0.660239983

0.406520034 0.521670000 0.688109970

0.395810012 0.282460006 0.501529980

0.226530003 0.115879996 0.501719999

0.392380004 0.113240005 0.493400002

0.119709994 0.227740006 0.503509998

0.286929982 0.396760003 0.506680012

0.116800006 0.393360008 0.500599957

0.895529965 0.282169996 0.502470016

0.727659993 0.114189996 0.503319979

0.896099976 0.114120005 0.504250002

0.615740025 0.223530003 0.499410009

0.782540018 0.391970016 0.502340031

0.614290016 0.390590012 0.495200014

0.392070009 0.784140019 0.496399975

0.225609995 0.613690041 0.498120022

0.394689996 0.617290022 0.498530006

0.112090041 0.724270031 0.496930027

0.280220022 0.892770048 0.494549990

0.112070018 0.892590037 0.497359991

0.889989964 0.779770057 0.505130005

0.721549975 0.611450012 0.506129980

0.889790066 0.612870006 0.496149969

0.610360031 0.723590017 0.509520006

0.780560032 0.888640023 0.506900024

0.616020036 0.894640068 0.500180006

0.496860023 0.563830038 0.521529961

0.508530018 0.486480012 0.602729988

0.399619984 0.592729991 0.642080021

0.488469994 0.425189991 0.695879984

0.446530023 0.562420032 0.735750008

0.326139984 0.452210013 0.702369976

0.342150002 0.607039984 0.655109978

Zn-CHO-CH_2_

1.0

14.2230997086 0.0000000000 0.0000000000

-7.1115498543 12.3175656682 0.0000000000

0.0000000000 0.0000000000 20.0000000000

C N Zn O H

26 24 1 1 3

Direct

0.222639993 0.443500015 0.508379984

0.064149995 0.285290006 0.505360031

0.223190000 0.285240009 0.504780006

0.281080003 0.063810000 0.500289965

0.440079987 0.221670009 0.494939995

0.283010011 0.223139999 0.502069998

0.719140021 0.441469998 0.495149994

0.562039967 0.282630010 0.493629980

0.721080000 0.284100000 0.500029993

0.783289982 0.065150000 0.500400019

0.941660005 0.223879998 0.504040003

0.783179979 0.223769998 0.501429987

0.219160001 0.941330026 0.500740004

0.060650005 0.782870034 0.502069998

0.219070009 0.782500025 0.500979996

0.282520021 0.564860009 0.508570004

0.442080019 0.728610029 0.506169987

0.280849998 0.723540001 0.501999950

0.721629977 0.944199976 0.500289965

0.560850011 0.788189987 0.505159950

0.720059976 0.783980061 0.504780006

0.779049965 0.563450003 0.496960020

0.938129991 0.721939958 0.502239990

0.779900045 0.722080018 0.503289986

0.470830009 0.455720027 0.657060051

0.375439996 0.450960004 0.670180035

0.391200028 0.281610027 0.496560001

0.223320013 0.114619999 0.504839993

0.389619987 0.113119998 0.495730019

0.114910006 0.227100001 0.503020000

0.282570026 0.394550014 0.507719994

0.114120001 0.393819984 0.508019972

0.892120035 0.283190017 0.504129982

0.724550069 0.115480005 0.499319983

0.892409977 0.115249998 0.502449989

0.612330063 0.224640010 0.498490000

0.778810007 0.392910020 0.500720024

0.610959998 0.391260000 0.489279985

0.389450009 0.783270006 0.501150036

0.224120004 0.613099994 0.503329992

0.393059996 0.617380027 0.512799978

0.109830015 0.723479997 0.501810026

0.277859990 0.891720025 0.500159979

0.110100013 0.891740077 0.501749992

0.888290031 0.780600042 0.506370020

0.719979985 0.613629999 0.498350000

0.887449954 0.613319994 0.497729969

0.610030000 0.726300009 0.509809971

0.778670021 0.890890040 0.499989986

0.613290042 0.895800033 0.500729990

0.499840002 0.564690025 0.535520029

0.535669976 0.499790036 0.603910017

0.503759995 0.420940037 0.692920017

0.331230032 0.413839992 0.716090012

0.341390016 0.486110003 0.635990047

Zn-CHO-CH_3_

1.0

14.2230997086 0.0000000000 0.0000000000

-7.1115498543 12.3175656682 0.0000000000

0.0000000000 0.0000000000 20.0000000000

C N Zn O H

26 24 1 1 4

Direct

0.224180007 0.443459987 0.509549999

0.065289987 0.284909990 0.504270029

0.224209996 0.284370016 0.506929970

0.282229996 0.062919997 0.500040007

0.441520018 0.221129997 0.497410011

0.283960023 0.222380006 0.504509974

0.720480054 0.441490012 0.497160006

0.563160011 0.282260001 0.495799971

0.722609949 0.283919989 0.500190020

0.784489983 0.064690005 0.501460028

0.943249950 0.223940001 0.502979994

0.784630017 0.223660017 0.501470041

0.220750014 0.940580020 0.500479984

0.062499995 0.782100053 0.500129986

0.221350002 0.781880014 0.503230000

0.283920011 0.563850014 0.510290051

0.444449967 0.728690008 0.508510017

0.282629982 0.723309974 0.504909992

0.723730020 0.944760061 0.501820040

0.562570002 0.788320059 0.507070017

0.723339970 0.784640023 0.505270004

0.780560040 0.563220054 0.498930025

0.940330020 0.721729984 0.499650002

0.782320060 0.723060050 0.503230000

0.470380014 0.453290000 0.649389982

0.380799990 0.471749993 0.669810009

0.392640021 0.281140006 0.502120018

0.224309992 0.113770001 0.504570007

0.390979987 0.112690004 0.495400000

0.116370003 0.226430013 0.503279972

0.284249983 0.393870000 0.512049961

0.115700015 0.393400036 0.506040001

0.893720041 0.283569994 0.502629995

0.725730016 0.115440006 0.500930023

0.894029949 0.115429999 0.502439976

0.613699979 0.224429998 0.499030018

0.780219961 0.392809989 0.501009989

0.611930026 0.391260000 0.492969990

0.391789985 0.782839994 0.504209995

0.226330000 0.612689995 0.506040001

0.395780019 0.616390007 0.514739990

0.111649970 0.722869974 0.502349949

0.279859982 0.891550002 0.502910042

0.111780008 0.891179993 0.498640013

0.890810003 0.780730037 0.502059984

0.721319970 0.613610024 0.502759981

0.888599985 0.612469995 0.497070026

0.612269975 0.725790017 0.510940027

0.780789962 0.891049998 0.500999975

0.614849945 0.895299952 0.502969980

0.506539980 0.566280002 0.520580006

0.523389994 0.485179986 0.596679974

0.494309996 0.408589992 0.684459972

0.306940017 0.392910020 0.678989983

0.364630033 0.518040018 0.632260036

0.402310022 0.514240051 0.718429995

Zn-CH_2_O-CH3

1.0

14.2230997086 0.0000000000 0.0000000000

-7.1115498543 12.3175656682 0.0000000000

0.0000000000 0.0000000000 20.0000000000

C N Zn O H

26 24 1 1 5

Direct

0.223739994 0.442259993 0.508239985

0.065119998 0.284110007 0.506500006

0.223959990 0.284029989 0.501039982

0.283050007 0.063470002 0.500570011

0.441679998 0.221470004 0.491690016

0.284300010 0.222450017 0.497640038

0.720880026 0.441300014 0.494219971

0.563700060 0.282599989 0.491219997

0.722479990 0.283880000 0.500509977

0.784099967 0.064379999 0.501900005

0.942619958 0.222980003 0.505739975

0.784259982 0.223210010 0.502880001

0.221389974 0.940970005 0.501909971

0.062820005 0.782480050 0.501349974

0.221200000 0.782050037 0.503240013

0.284139995 0.563790049 0.509410000

0.443569998 0.727020051 0.506860018

0.282699991 0.722710016 0.504489994

0.722650015 0.943350016 0.501550007

0.562300023 0.786660013 0.505420017

0.721780010 0.783249953 0.505069971

0.781050048 0.563200001 0.496099997

0.940270077 0.721760025 0.500969982

0.781930034 0.721820028 0.503109980

0.488620033 0.482320024 0.665920019

0.377290020 0.472790029 0.673829985

0.392120002 0.280810006 0.489369965

0.225229989 0.114280002 0.504040003

0.391649989 0.113110000 0.496230030

0.115570003 0.225850011 0.501679993

0.283349991 0.393330006 0.503250027

0.115319994 0.392580001 0.510999966

0.893220022 0.282440011 0.505919981

0.725480056 0.114880008 0.500689983

0.893210001 0.114399999 0.504010010

0.613800009 0.224610009 0.497530031

0.780239971 0.392640004 0.501630020

0.612990055 0.391229998 0.486439991

0.391160018 0.782140004 0.503269958

0.225700016 0.612270009 0.505840015

0.394740006 0.615860002 0.512209988

0.111970010 0.723090013 0.502210045

0.280039998 0.891290012 0.503550005

0.112349999 0.891360003 0.500819969

0.890410036 0.780340052 0.505729961

0.721959991 0.613340047 0.498350000

0.889470014 0.613129995 0.496049976

0.611580017 0.724780022 0.509190035

0.780030022 0.890320045 0.501550007

0.614279977 0.894310010 0.501559973

0.503369981 0.561919990 0.534669971

0.518760032 0.478140022 0.598890018

0.488669977 0.414390012 0.692960024

0.313279981 0.396600006 0.651809978

0.375319978 0.541019981 0.648850012

0.358390024 0.474940014 0.727029991

0.550170011 0.558060020 0.690939999

Zn-CH_2_OH-CH_3_

1.0

14.2230997086 0.0000000000 0.0000000000

-7.1115498543 12.3175656682 0.0000000000

0.0000000000 0.0000000000 20.0000000000

C N Zn O H

26 24 1 1 6

Direct

0.222490009 0.446530039 0.502290010

0.065190004 0.286639989 0.498899984

0.225940011 0.289150014 0.497779989

0.280649982 0.065580003 0.504980040

0.441289996 0.221740020 0.506040001

0.285670023 0.226770001 0.498250008

0.721820012 0.441630033 0.514050007

0.562390043 0.280350011 0.510299969

0.722510027 0.283930016 0.500220013

0.784079993 0.064960006 0.499590015

0.942930023 0.224370014 0.499060011

0.784210052 0.224040014 0.498589993

0.217699986 0.943559990 0.504869986

0.059520017 0.785130039 0.506690025

0.218250016 0.785040072 0.502670002

0.280079993 0.567070036 0.504620028

0.441069994 0.732280001 0.504469967

0.279380020 0.726470032 0.501150036

0.722050052 0.944780036 0.500869989

0.559420035 0.789980028 0.504699993

0.718230023 0.782850059 0.507269955

0.779810009 0.563170038 0.513030005

0.937619931 0.723090013 0.508790016

0.777729996 0.721180042 0.508520031

0.501600049 0.472340002 0.654439974

0.398140003 0.471779995 0.674040031

0.394670009 0.283520017 0.498000002

0.224690002 0.118409997 0.500629997

0.389209965 0.113530006 0.510420036

0.117830008 0.229960002 0.496000004

0.284460021 0.398880025 0.501079988

0.114060013 0.395080019 0.502040005

0.893400045 0.283910021 0.498519993

0.725379997 0.115700005 0.498930025

0.893800000 0.115720000 0.499760008

0.613200034 0.225030014 0.498519993

0.779640028 0.393209999 0.502390003

0.613109983 0.388240003 0.524440002

0.388489999 0.786530018 0.500209999

0.222329994 0.615700005 0.500619984

0.391020008 0.620369984 0.510179996

0.108869979 0.726069982 0.504809952

0.276750000 0.894490021 0.502719975

0.108630020 0.894330063 0.506829977

0.886059994 0.780279971 0.506770039

0.718550043 0.611680000 0.511219978

0.887890007 0.613960057 0.512589979

0.606970008 0.725520040 0.510730028

0.777350013 0.889400017 0.502569962

0.613240016 0.896899995 0.500220013

0.492820019 0.566519978 0.523360014

0.506910036 0.468540036 0.582560015

0.502240015 0.400400011 0.674170017

0.326279986 0.397290008 0.656499958

0.396409991 0.541890033 0.651929951

0.393450000 0.477290024 0.728620005

0.573959993 0.545640022 0.673520041

0.553570002 0.436720040 0.561140013
